# Unlocking conserved and diverged metabolic characteristics in cassava carbon assimilation via comparative genomics approach

**DOI:** 10.1038/s41598-018-34730-y

**Published:** 2018-11-09

**Authors:** Wanatsanan Siriwat, Saowalak Kalapanulak, Malinee Suksangpanomrung, Treenut Saithong

**Affiliations:** 1 Systems Biology and Bioinformatics Research Laboratory, Pilot Plant Development and Training Institute, King Mongkut’s University of Technology Thonburi, Bang Khun Thian, Bangkok 10150 Thailand; 2 Bioinformatics and Systems Biology Program, School of Bioresources and Technology, King Mongkut’s University of Technology Thonburi, Bang Khun Thian, Bangkok 10150 Thailand; 3 National Center for Genetic Engineering and Biotechnology, Pathum Thani, 12120 Thailand

## Abstract

Globally, cassava is an important source of starch, which is synthesized through carbon assimilation in cellular metabolism whereby harvested atmospheric carbon is assimilated into macromolecules. Although the carbon assimilation pathway is highly conserved across species, metabolic phenotypes could differ in composition, type, and quantity. To unravel the metabolic complexity and advantage of cassava over other starch crops, in terms of starch production, we investigated the carbon assimilation mechanisms in cassava through genome-based pathway reconstruction and comparative network analysis. First, MeRecon — the carbon assimilation pathway of cassava was reconstructed based upon six plant templates: Arabidopsis, rice, maize, castor bean, potato, and turnip. MeRecon, available at http://bml.sbi.kmutt.ac.th/MeRecon, comprises 259 reactions (199 EC numbers), 1,052 proteins (870 genes) and 259 metabolites in eight sub-metabolisms. Analysis of MeRecon and the carbon assimilation pathways of the plant templates revealed the overall topology is highly conserved, but variations at sub metabolism level were found in relation to complexity underlying each biochemical reaction, such as numbers of responsible enzymatic proteins and their evolved functions, which likely explain the distinct metabolic phenotype. Thus, this study provides insights into the network characteristics and mechanisms that regulate the synthesis of metabolic phenotypes of cassava.

## Introduction

Metabolism is the foundation of cellular regulation in living organisms. It is where biological regulatory factors express their modulating actions in pursuit of normal cellular activities, resulting in the diverse survival behaviors observed in various environments^1^. Primary metabolic pathways for cellular survival, such as those involved in carbon assimilation, are typically conserved across taxa^1,2^. The conservation of these pathways is high among organisms within a kingdom, e.g. Archaebacteria, Animalia, and Plantae^3^ as well as across ancestral kingdoms. Preservation of metabolic pathways is evidenced by heredity in organisms that share evolutionary lineage^1,2^. With these highly conserved metabolic pathways, similarities in the metabolic processes as well as the overall context of metabolism (i.e., types of metabolic compounds within the cell) are expected. Despite the conserved metabolic pathways, some evolutionarily related organisms have different spectrum of metabolic compounds, especially regarding their accumulated metabolites. For example, phyto-products within crops of the Euphobiaceae family include: starch from cassava (*Manihot esculenta* Crantz), latex rubber from rubber tree (*Hevea brasiliensis*), and triacylglycerols (TAGs) from physic nut (*Jatropha curcas* L.)^3^.

Cassava is an important source of calories, especially for poor households in the tropics. Its starch is synthesized and stored in underground roots making up 70–85 percent of the total dry weight of cassava roots^4^, with very low protein and lipid contents compared to cereal grains^5–7^. Although the metabolic pathway of starch biosynthesis is highly conserved amongst starch crops^8^, starch yield varies. For example, cassava yields more starch in comparison with rice, maize, and potato. To gain insight into the complexity of metabolism and unravel the metabolic advantage of cassava over other starch crops in terms of starch production, direct comparison of the constituent metabolic reactions is insufficient. In addition, there is need to understand the enzymatic proteins involved as well as their biochemical properties and functions.

Comprehensive understanding of interspecies differences in metabolism extends beyond the knowledge about individual genes and gene functions. The metabolic processes underlying metabolism involve about 15–20 percent of protein-coding genes in genomes, related to thousands of enzymes in the metabolic pathways^9^. The cooperative role of these genes is represented by the metabolic network, the series of biochemical reactions describing the conversion of substrates to products of metabolism^10,11^. The large number of genes governing the metabolic processes introduces complexity into the metabolic network, of which metabolic flexibility^12^ and plasticity^13^ due to redundancy in reactions and metabolic paths are indicative^14^. Thus, system-level analysis is crucial to comprehensively study metabolic processes in living organisms; it might be the only way to access the hidden factors behind the distinct metabolic characteristics of organisms^13^.

Advances in high-throughput sequencing technology have enabled studies on the global metabolic capabilities that help define metabolism. The exponential growth of genome sequencing projects in the last decades^15^, and the availability of complete genome sequences for a number of organisms have provided a plethora of resources for comparative genomics studies^16^, and have made finding results more promising^11^. These resources have facilitated the large-scale reconstruction of metabolic pathways for a wide range of species, especially for non-model organisms^10,17^. The biochemical reactions in cellular metabolism are inferred from information in publicly available databases: species-specific databases^18,19^ and metabolic pathway omnibus databases such as KEGG (Kyoto Encyclopedia of Genes and Genomes)^20^, PMN (Plant Metabolic Network)^21^, MetaCyc^22^, and BioCyc^22^. Genome-scale metabolic pathway reconstruction enhances our understanding of metabolic characteristics and capabilities.

The comparison of reconstructed metabolic pathways could reveal conserved as well as diverged metabolic pathways linked to distinct metabolic phenotypes in closely related species. The findings would highlight the crucial metabolic paths which are conserved in various species throughout the phylogenetic lineage as well as underline the special paths required to retain the specific metabolic activity of species^1,23,24^. For example, the comparative analysis of four halophilic archaea species: *Halobacterium salinarum*, *Haloarcula marismortui*, *Haloquadratum walsbyi*, and *Natronomonas pharaonis* demonstrated similarities in nucleotides and prenyl-based lipids synthesis and differences in glycerol degradation, pentose metabolism, and folate synthesis^25^. Furthermore, metabolic routes for acetyl-CoA biosynthesis related to lipid production are present in oleaginous fungi (*Yarrowia lipolytica*, *Rhizopus oryzae*, *Aspergillus oryzae*, and *Mucor circinelloides*), but are absent in non-oleaginous strains (*Saccharomyces cerevisiae*, *Candida albicans*, and *Ashbya gossypii*) as determined by comparative analysis^26^. In addition to these metabolic paths, the characteristics of metabolism may also be explained by the number of enzymatic protein isoforms (multiple proteins that perform the same function), heteromeric enzyme complexes (more than one subunit required for carrying out the enzymatic function), and enzymes that catalyze several reactions which determine the flexibility of metabolic conversion of substrates to products^27^. While there is no absolute way for investigating metabolism, detailed study including extensive features would help to decode the metabolic characteristics underlying the distinct behaviors.

To examine the metabolic characteristics of cassava that facilitate starch production, we reconstructed the carbon assimilation pathway using a comparative genomic approach based on multiple template plants proposed by Saithong *et al*.^8^ with the inclusion of turnip — a starchy root crop as cassava, to enhance the protein annotation. In order to reflect the complexity of carbon metabolism in cassava, the pathways of amino acid, cell wall, fatty acid, and nucleotide biosynthesis and respiration were incorporated into MeRecon in addition to those of starch and sucrose biosynthesis and the Calvin cycle as contained in Saithong *et al*.^8^. Following the manual curation and validation with available transcriptome data and literature, the starch biosynthesis, sucrose biosynthesis, and Calvin cycle pathways in MeRecon were enhanced their quality in comparison to Saithong *et al*.^8^, and the overall network completeness is better in MeRecon than CassavaCyc. Analysis of MeRecon pathway allowed us to characterize the complexity of carbon metabolism in cassava in comparison to the template plants. The results revealed that although the overall topology was highly conserved, differences at sub-metabolism level were found, especially in non-carbohydrate related pathways. In addition, our study showed that some enzymatic proteins of cassava evolved from their ancestral orthologues, as exemplified by phosphoenolpyruvate synthase (EC 2.7.9.2; PEPS) and aldehyde dehydrogenase (NAD^+^) (EC 1.2.1.3; ALDH), which could explain the distinct metabolic pathway of cassava and might result in functional adaptation of enzymatic proteins.

## Methods

### Reconstruction of the metabolic pathway

**Figure 1:**
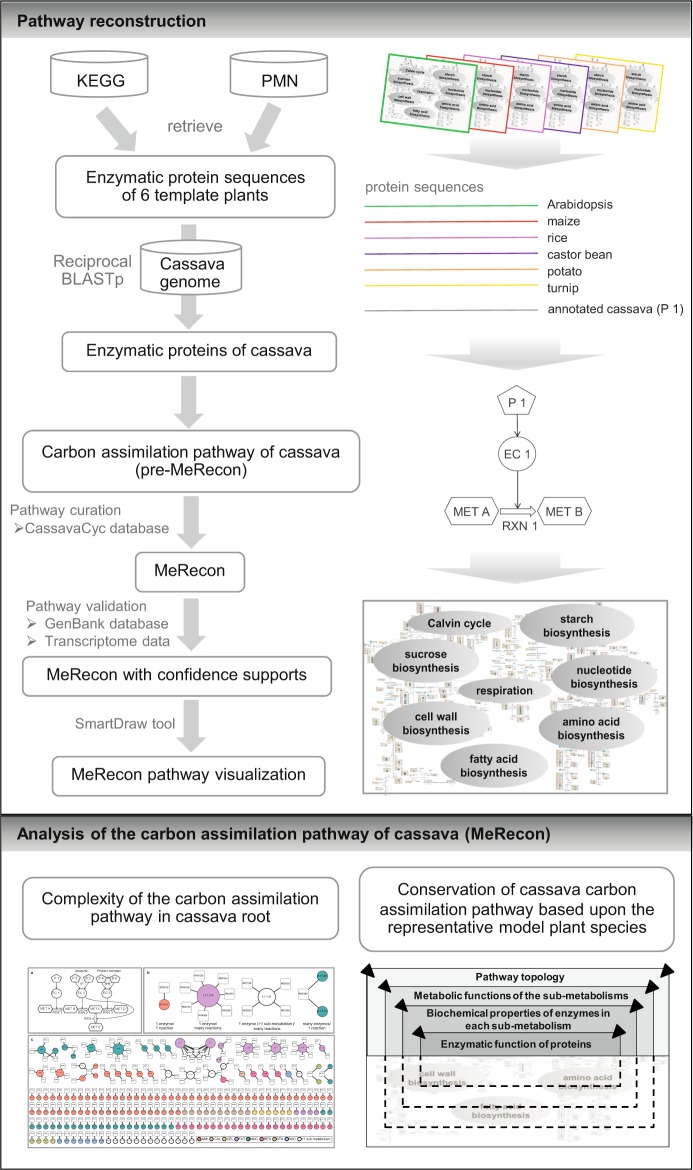
Overview of the methodology. There are two main parts: pathway reconstruction, based on the protocol of Saithong *et al*.^8^, and analysis of the reconstructed metabolic pathway. KEGG database - Kyoto Encyclopedia of Genes and Genomes^20^ and PMN database - Plant Metabolic Network^21^.

The carbon assimilation pathway of cassava (MeRecon) was reconstructed based upon the plant genomic data from various databases^28^ (Supplementary Table S1), and the metabolic pathway information from KEGG^20^ and PMN databases^21^. MeRecon consists of eight sub-metabolisms: Calvin cycle, sucrose biosynthesis, starch biosynthesis, respiration, amino acid biosynthesis, cell wall biosynthesis, fatty acid biosynthesis, and nucleotide biosynthesis. The pathway was reconstructed according to the genome-based approach proposed by Saithong *et al*.^8^. The methodology, in brief, is presented in Fig. 1. First, cassava genes relevant to the carbon assimilation pathway were annotated based upon their orthologues in six template plants including turnip (*Brassica rapa*) — a starchy root crop, Arabidopsis (*Arabidopsis thaliana*) — a model plant, maize (*Zea mays*) — a C4-starchy cereal crop, rice (*Oryza sativa*) — a C3-starchy cereal crop, castor bean (*Ricinus communis*) — an oil-seed crop of the Euphorbiaceae family, and potato (*Solanum tuberosum*) — a starchy tuber crop (Table S1). The annotation was performed by reciprocal BLASTp with the following criteria: E-value ≤ 1 × 10^−10^, identity percentage ≥60, and coverage percentage ≥80. Next, conservation score (*CS*) and match score (*MS*)^8^, were calculated for each annotated protein to represent the confidence level. The preliminarily reconstructed carbon assimilation pathway, pre-MeRecon, was curated with the biochemical reaction obtained from the CassavaCyc database (http://www.plantcyc.org/). Following curation, MeRecon was visualized using SmartDraw, an informative graphical platform, to reveal the constituent metabolites, reactions, protein IDs with the *CS* and *MS* scores, and the template plants from which the cassava orthologues were identified.

### Validation of the metabolic pathway

Here, cassava genes annotated to the carbon assimilation pathway (MeRecon) were verified using cassava cloned sequences from GenBank database^29^ and gene expression data from six transcriptome datasets on: leaf and stem^30^, root development^30–32^, cold stress^33^, drought stress^34^, and fungal infection^35^.

### Analysis of metabolic pathway

MeRecon was comparatively analyzed with the existing carbon assimilation pathways of turnip, Arabidopsis, maize, rice, castor bean, and potato based upon their evolutionary and physiological relatedness to cassava, availability of well-defined genome information, and being starch crops. The overall pathway topology, metabolic functions of the sub-metabolisms, biochemical properties of enzymes in each sub-metabolism, and enzymatic function of proteins were examined to identify conserved and diverged enzymatic proteins (Fig. 1). The normalized Hamming distance (*HD*) was calculated for each sub-metabolism to measure the distance between cassava and the templates, based on the co-existence of reactions in each sub-metabolism. Additionally, the biochemical properties of the enzymes were determined by calculating the *P* score as shown below.

#### Normalized Hamming distance (HD)

For the *j* ϵ {*Calvin cycle*, *sucrose biosynthesis*, *starch biosynthesis*, *respiration*, *amino acid biosynthesis*, *cell wall biosynthesis*, *fatty acid biosynthesis*, *nucleotide biosynthesis*} pathway, *HD* estimates the similarity of pathway topology according to the co-existence (*a*_*i*_) of the enzymatic reaction *i* (1, 2, 3, …, *I*) in cassava (MeRecon) and the *k* ϵ {*turnip, Arabidopsis, maize, rice, castor bean, potato*} plant species. *HD* was calculated as follows:

1 $$H{D}_{j}^{k}=\frac{{\sum }_{i=1}^{I}{a}_{i}}{{b}_{j}}$$

where

*a*_*i*_ = co - existence of reaction *i* in pathway *j* of cassava and species *k*

*a*_*i*_ = {0, *reaction i occurs in both organisms*1, *otherwise*

*b*_*j*_ = the number of enzymatic reactions in pathway *j*

#### P score

The *P* score of the *j* ϵ {*Calvin cycle*, *sucrose biosynthesis*, *starch biosynthesis*, *respiration*, *amino acid biosynthesis*, *cell wall biosynthesis*, *fatty acid biosynthesis*, *nucleotide biosynthesis*} pathway estimates the similarity of biochemical properties of enzymatic functions (EC number) *l* (1, 2, 3, …, *L*) between cassava (MeRecon) and the *k* ϵ {*turnip, Arabidopsis, maize, rice, castor bean, potato*} plant species as follows:

2 $$P\frac{k}{j}=\frac{{\sum }_{l=1}^{L}\frac{{c}_{l}}{{C}_{P/E,l}}}{{d}_{j}}$$

where *C*_*P/E, l*_ = the number of cassava enzymatic proteins annotated to an enzyme (EC number) according to all template plant species.

*       c*_*l*_ = the number of cassava enzymatic proteins annotated from the *k* plant species in sub-metabolism *j*.

*       d*_*j*_ = the number of enzymes (EC number) in pathway *j*.

### Phylogenetic tree analysis of the enzymatic protein sequences

The phylogenetic trees were constructed using the ClustalW alignment of the MEGA7 program^36^ with default parameters. The maximum likelihood (ML) tree was constructed based on the Jones-Taylor-Thornton (JTT) amino acid substitution model to determine the evolutionary relatedness of the protein sequences. Bootstrap values were also applied with 100 replicates.

### Conserved domain analysis of annotated enzymatic proteins

The amino acid sequences of cassava annotated to phosphoenolpyruvate synthase (EC 2.7.9.2) and their orthologues in two template plants (maize and rice), and other species including moss (representative of primitive plants), Chinese alligator (representative of animals), *Escherichia coli* (representative of bacteria), and red algae (representative of algae) were retrieved from KEGG database^20^ and analyzed to identify conserved protein domains using InterPro^37^ (https://www.ebi.ac.uk/interpro/) with default parameters.

The amino acid sequences of cassava annotated to aldehyde dehydrogenase (NAD+) (EC 1.2.1.3; ALDH) and amino acid sequences of the six template plants were analyzed using the Batch CD-Search Tool^38^ (https://www.ncbi.nlm.nih.gov/Structure/bwrpsb/bwrpsb.cgi). To identify the conserved protein domain superfamilies by comparing the protein query sequence against the Conserved Domain Database (CDD) with the parameters set as follows: E-value of 0.01, composition-corrected scoring applied with the low complexity region turned off, and maximum number of hits of 500.

Finally, the iTOL (interactive tree of life) program^39^ (https://itol.embl.de/) was employed to integrate the phylogenetic tree and protein domains.

## Results and Discussion

### Genome-based pathway reconstruction of carbon assimilation (MeRecon): the backbone metabolic network of carbon conversion towards starch biosynthesis in cassava roots

The study aimed to gain more understanding into the mechanism by which starch is synthesized from photo-assimilated carbon in cassava and stored in underground roots. The carbon assimilation pathway that describes the synthesis of starch in cassava root was reconstructed following comparative genomics approach^8^. Protein sequences of enzymes in the carbon assimilation pathway from multiple well-studied plants (maize, potato, rice, Arabidopsis and castor bean) were compared against all protein sequences in the cassava genome to identify genes that are conserved among the species, as well as genes that give cassava its unique characteristics. In addition, turnip was also employed as representative template of starchy root crops to exhaustively identify the genes that are related to carbon assimilation in cassava. The preliminarily reconstructed carbon assimilation pathway of cassava, pre-MeRecon, comprised eight sub-metabolisms: Calvin cycle, starch biosynthesis, sucrose biosynthesis, respiration, amino acid biosynthesis, nucleotide biosynthesis, fatty acid biosynthesis, and cell wall biosynthesis and contained 259 reactions—253 enzymatic reactions, two spontaneous reactions, and four gap reactions (three gap reactions in amino acid biosynthesis and one gap reaction in nucleotide biosynthesis), 259 metabolites, and 864 metabolic genes corresponding to 1,046 enzymatic proteins (pre-MeRecon, Table 1). Gap reactions refer to reactions of the inferred pathway with missing enzymatic proteins required for completing the biosynthesis pathway of metabolic products. The proteins could not be annotated probably because the orthologous genes were not found in the template plants.

**Table 1:** Characteristics of the reconstructed carbon assimilation pathway of cassava for pre-MeRecon and MeRecon, including EC numbers, enzymatic reactions, spontaneous reactions, gap reactions, genes, proteins, and metabolites in each sub-metabolism.

Sub-metabolisms	pre-MeRecon								MeRecon							
	EC numbers	Reactions				Genes	Proteins	Metabolites	EC numbers	Reactions				Genes	Proteins	Metabolites
		Enzymatic reactions^a^	Spontaneous reactions^b^	Gap reactions^c^	Total					Enzymatic reactions^a^	Spontaneous reactions^b^	Orphan reactions^d^	Total			
Calvin cycle	21	23	—	—	23	128	158	35	21	23	—	—	23	128	158	35
Sucose biosynthesis	15	16	—	—	16	64	79	25	15	16	—	—	16	64	79	25
Starch biosynthesis	13	14	—	—	14	82	106	19	13	14	—	—	14	82	106	19
Respiration	36	40	—	—	40	262	325	49	36	40	—	—	40	262	325	49
Amino acid biosynthesis	76	87	2	3	89	250	304	121	78	89	2	1	92	254	308	122
Cell wall biosynthesis	6	6	—	—	6	85	91	15	6	6	—	—	6	85	91	15
Fatty acid biosynthesis	12	35	—	—	35	59	65	49	12	35	—	—	35	59	65	49
Nucleotide biosynthesis	34	51	—	1	51	123	146	67	35	52	—	—	52	125	148	67
Total	196	253	2	4	259	864	1,046	259	199	256	2	1	259	870	1,052	259

^a^Enzymatic reactions are catalyzed by enzymes to perform their functions.

^b^Spontaneous reactions can perform functions automatically without regulatory enzymes.

^c^Gap reactions are reactions that require enzymatic proteins for producing essential components, but their encoding genes could not be identified.

^d^Orphan reactions are reactions of which the related enzymatic genes have not been yet identified, but have been biochemically characterized.

Subsequently, to ensure a gap-free network, pre-MeRecon was curated with the CassavaCyc database (http://plantcyc.org/), as shown in Supplementary Table S2. The results show that pre-MeRecon covered the carbon assimilation pathway of CassavaCyc with at least 76 percent overlap of reactions. Three gap reactions in pre-MeRecon, consisting of two gap reactions in the amino acid biosynthesis pathway and one gap reaction in the nucleotide biosynthesis pathway, were curated using reactions: R03013, R03508, and R04591 that were obtained from CassavaCyc. Gaps in the amino acid biosynthesis pathway were filled with four proteins and four genes, whereas the gap in nucleotide biosynthesis pathway was filled with two proteins and two genes (Table 1). The remaining gap reaction of which the enzymatic protein was not found in CassavaCyc, i.e. orphan reaction, was included to fully connect the amino acid biosynthesis pathway. The pre-MeRecon contained 42 enzymes (EC numbers) and 65 reactions that are not found in CassavaCyc. These reactions were reconstructed with 217 proteins corresponding to 179 genes identified in our study (Supplementary Table S3). The expression data on leaf and stem^30^, root development^30–32^, cold stress^33^, drought stress^34^, and fungal infection^35^ were applied to support the existence of the reactions in MeRecon (Supplementary Table S3). Overall, pre-MeRecon was curated using four reactions related to 17 metabolites. MeRecon, thus, consists of 259 reactions relevant to 199 EC numbers corresponding to 1,052 enzymatic proteins (870 genes) and 259 metabolites (Tables 1 and Table S4). The MeRecon pathway is available at http://bml.sbi.kmutt.ac.th/MeRecon.

**Figure 2:**
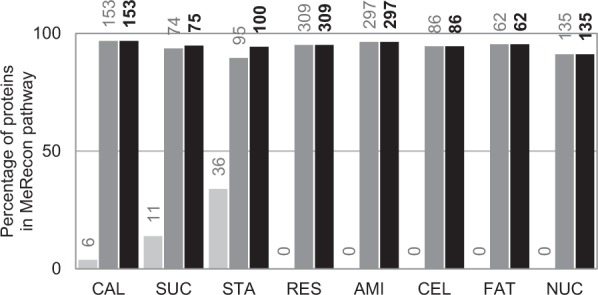
The percentage of proteins in the cassava carbon assimilation pathway (MeRecon) supported by cloned sequences and gene expression datasets: light gray - cloned sequences reported in GenBank database, dark gray - expression evidence in at least one dataset, and black - total number of sequences supported by data on cloned sequences and/or transcript sequences. The numbers at the top of each bar show the number of proteins. The six transcriptome datasets on: leaf and stem^30^, root development^30–32^, cold stress^33^, drought stress^34^, and fungal infection^35^ were employed in this analysis. CAL - Calvin cycle, SUC - sucrose biosynthesis, STA - starch biosynthesis, RES - respiration, AMI - amino acid biosynthesis, CEL - cell wall biosynthesis, FAT - fatty acid biosynthesis, and NUC - nucleotide biosynthesis.

The curated pathway, MeRecon, was validated using either the cloned sequences reported in GenBank database^29^ or the expressed transcripts obtained from the six transcriptome datasets of cassava^30–35^ or both when available. The genes and proteins that were annotated as enzymes in MeRecon, based on comparative genomics approach, were verified for their existence in cassava metabolism. From 1,052 annotated proteins in MeRecon, 44 unique proteins (~4%) in three of the eight sub-metabolisms were supported by cloned sequences of cassava with exact EC numbers (Fig. 2, light-gray bars). Most of the verified proteins were related to starch biosynthesis (36 of 44) and accounted for 34 percent (36/106) of proteins in starch biosynthesis pathway of MeRecon, reflecting the cassava cloned sequences in GenBank database. The collection of cassava cloned sequences in GenBank covers only 10 percent (3,408/34,151) of the entire cassava genome (version 4.1)^40^ most of which are possibly involved in starch biosynthesis, the most studied metabolic pathway in cassava.

To further consolidate MeRecon, the annotated proteins of relevance were examined for their expression evidence observed in all six aforementioned transcriptome datasets^30–35^. The results show that 998 of 1,052 proteins exhibited expression activity at the gene level in cassava, from which 653 proteins (~65%) were confirmed by multiple transcriptome datasets (Supplementary Fig. S1). These expression data supported nearly 95 percent of all proteins in MeRecon, corresponding to: 153 of 158 proteins (97%) in Calvin cycle, 297 of 308 proteins (96%) in amino acid biosynthesis, 309 of 325 proteins (95%) in respiration, 62 of 65 proteins (95%) in fatty acid biosynthesis, 86 of 91 proteins (95%) in cell wall biosynthesis, 74 of 79 proteins (94%) in sucrose biosynthesis, 135 of 148 proteins (91%) in nucleotide biosynthesis and 95 of 106 proteins (90%) in starch biosynthesis sub-metabolisms (Fig. 2, dark gray bars). In summary, more than 95 percent of proteins in MeRecon (1,003 of 1,052) were validated using transcriptome data or GenBank information or both depending on their availability (Fig. 2, black bars), indicating its robustness and reliability.

**Figure 3:**
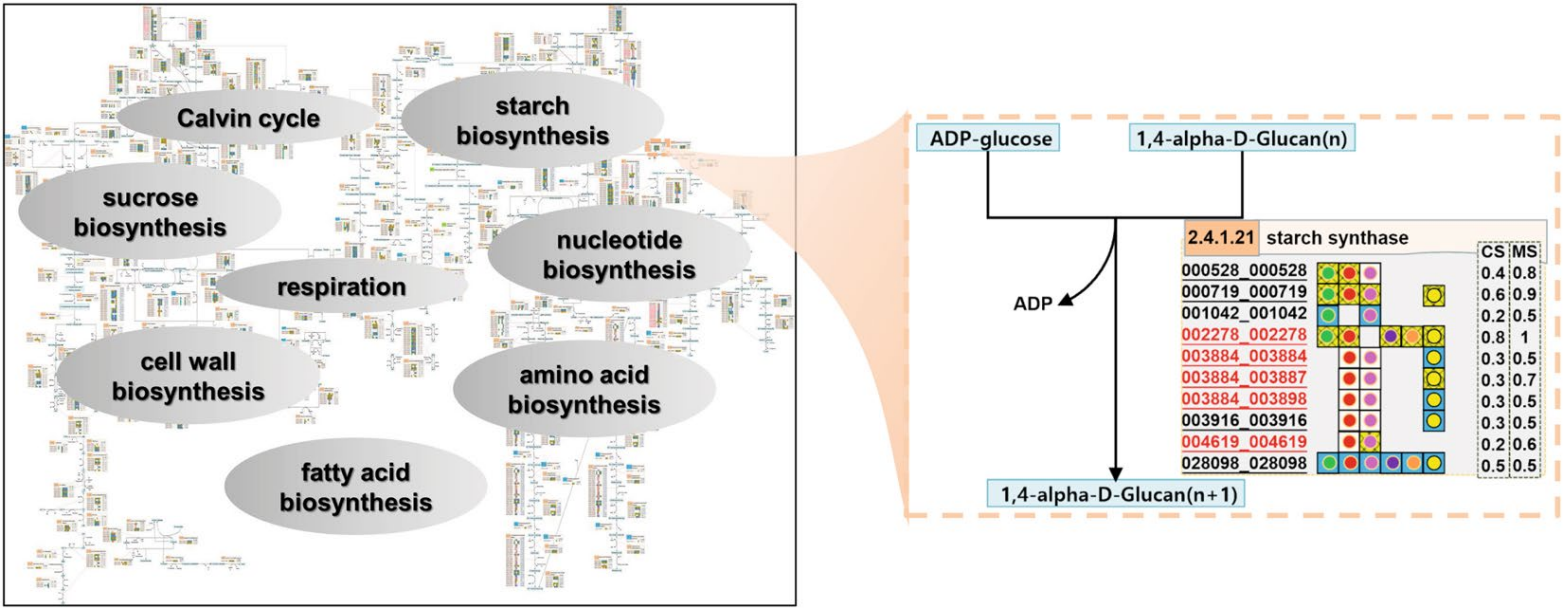
The visualization of the reconstructed carbon assimilation pathway (MeRecon). The approach is based on Saithong *et al*.^8^, and the pathway shows the relationship of metabolites, enzymatic reactions, enzymes — represented as EC numbers, enzyme functions, and gene IDs and protein IDs — both IDs designated as 12-digits code. The red colored codes indicate annotated proteins that were supported by cloned sequences in GenBank, while underlined codes indicate those supported by expression datasets. The conservation score (*CS*) and match score (*MS*) indicate the confidence level of the annotated proteins. The template plants from which the relevant cassava proteins were annotated, are denoted by colored circles:  Arabidopsis,  maize,  rice,  castor bean,  potato, and  turnip. The color of background grids represents the quality gradient of sequence alignment: yellow – high value, blue – mid value, and white – low value.

The confidence scores, consisting of conservation score (*CS*) and match score (*MS*), were assigned to each annotated protein in MeRecon based on the method of Saithong *et al*.^8^. The *CS-MS* plot (Supplementary Fig. S2) shows the confidence of all annotated proteins in MeRecon pathway. Majority of the annotated proteins had high *MS* scores with varying levels of *CS* scores. Although some annotated proteins had low *CS* scores, they were supported by cloned sequences from GenBank database and gene expression datasets, as denoted by red circle and green diamond, respectively (Supplementary Fig. S2). Finally, The carbon assimilation metabolic pathway of cassava (MeRecon) was visualized in the same manner as Saithong *et al*.^8^ to reveal pathway components (Figs 3 and S3–S10). Sets of proteins were annotated to the relevant metabolic reaction in MeRecon. The annotated proteins were represented by 12-digits IDs, which were formulated by combining the Phytozome IDs of genes and the corresponding proteins. The proteins, *CS-MS* scores, cloned sequences (red), and expression data (underlined) were used to indicate the confidence of annotation.

Furthermore, the complexity and conservation characteristics of the carbon assimilation pathway (MeRecon) were investigated. The complexity was analyzed based on the level of metabolic redundancy, i.e., the relationship between metabolic reactions and their corresponding enzymatic proteins, as multiple proteins catalyze each reaction in cassava pathway. The conservation was studied by comparing MeRecon with the corresponding pathways in the representative plants. Comparative analysis of the metabolic pathway was performed to demonstrate the conservation of carbon assimilation between cassava and the other six template plants, in terms of pathway structures and metabolic functions.

### Complexity of the carbon assimilation pathway in cassava root

**Figure 4:**
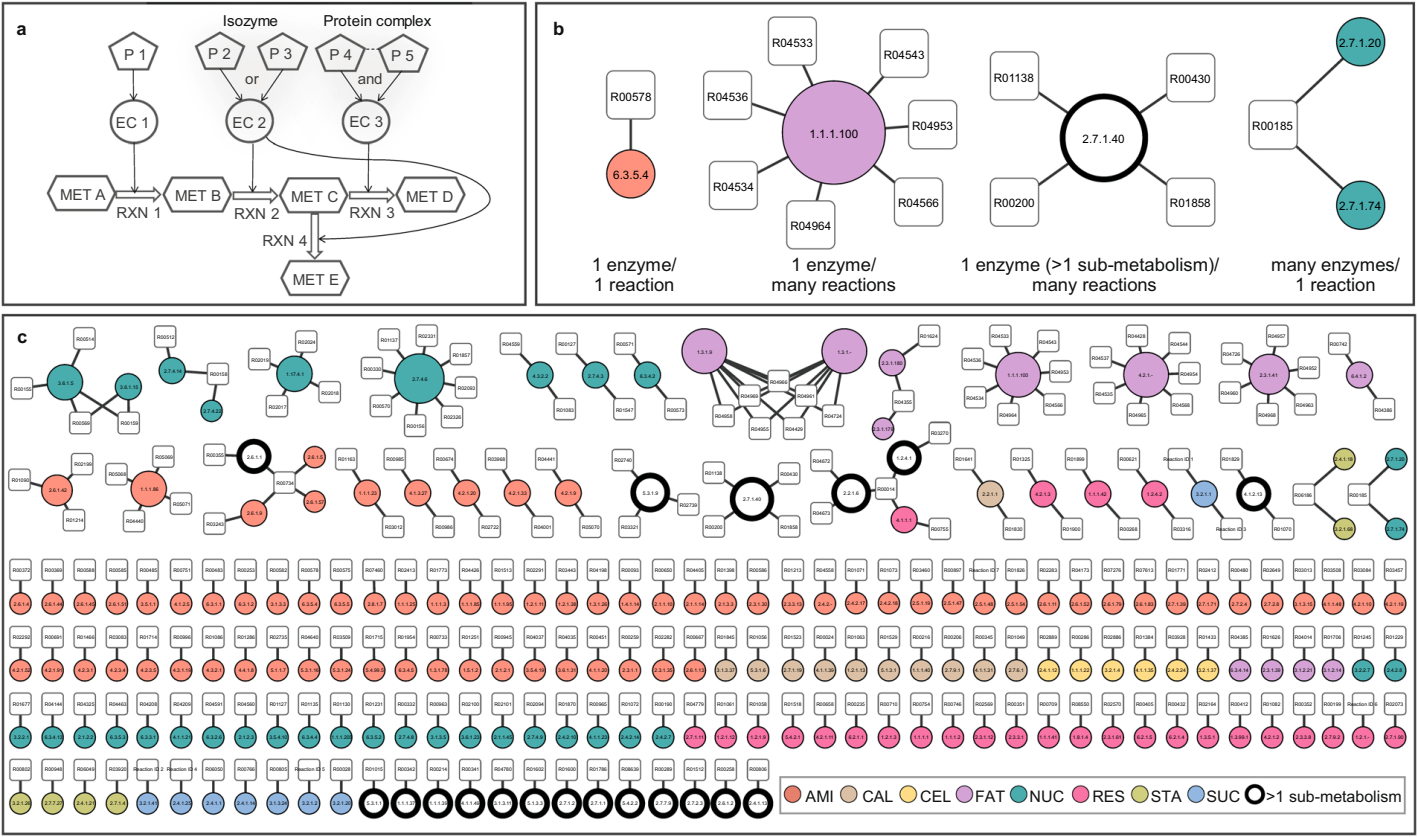
The complexity of the carbon assimilation metabolic pathway of cassava based on reaction-enzyme association. (**a**) The schematic of relationship among annotated enzymatic proteins (P), enzymes (EC), enzymatic reactions (RXN), and metabolites (MET); (**b**) Examples of the reaction-enzyme association in MeRecon; (**c**) The relationship between enzymes (EC numbers) and reactions in MeRecon, represented as nodes and edges. Nodes consist of enzymes (circles) and enzymatic reactions (rectangles), and the edges between the nodes represent reaction-enzyme association of each metabolic reaction. Size of enzyme nodes represents node degree, and the number of enzymatic reactions catalyzed by that enzyme. AMI - amino acid biosynthesis, CAL - Calvin cycle, CEL - cell wall biosynthesis, FAT - fatty acid biosynthesis, NUC - nucleotide biosynthesis, RES – respiration, STA - starch biosynthesis, and SUC - sucrose biosynthesis.

Complexity of the metabolic pathways in living organisms has been demonstrated in many studies^14,41^. The observed complexity could be due to the extensive number of metabolic components (e.g., reactions, enzymes, proteins, and metabolic compounds) or their complicated association in cellular metabolism. For a metabolic process, biochemical reactions not only link up in a sequential manner, but also are interconnected through common metabolic intermediate compounds. Enzymes are typically required to convert substrates to products in a biochemical reaction, but the enzyme-to-reaction proportion often varies. Some enzymes with different amino acid sequences are often involved in the same biochemical reaction (isozymes) with overlapped function, e.g., acid phosphatase (ACP; EC 3.1.3.2), esterases (EST; EC 3.1.1.1), malate dehydrogenase (MDH; EC 1.1.1.37), and shikimate dehydrogenase (SKDH; EC 1.1.1.15) isozymes in cassava^42^. In addition, enzymatic proteins often necessitate intricate regulatory pathway to pursue their normal function^9^, such as the heteromeric enzyme complex (protein complex). The complication, thus, would even increase for enzymes that require multiple amino acid sequences to perform their catalytic activity. These scenarios introduce flexibility and complexity into the carbon metabolic network (Fig. 4A). In this work, we employed the knowledge obtained from MeRecon to study the complexity of carbon assimilation pathway of cassava. The complexity of the metabolic process was first investigated through the reaction-enzyme relationship and then, examined through the number of proteins annotated to enzymes Fig. 4A.

The relationship between reactions (denoted by reaction IDs; square) and enzymes (denoted by EC number; circle) in MeRecon included: 1 enzyme/1 reaction, 1 enzyme/many reactions, 1 enzyme (>1 sub-metabolism)/many reactions, and many enzymes/1 reaction (Fig. 4B,C), an indication of the complicated reaction-enzyme association in the cassava carbon assimilation pathway. The size of enzyme node represents number of reactions associated with that enzyme, and the colors code indicates the functional pathway of the enzyme. Nonetheless, majority of the enzymes in MeRecon catalyzed a particular metabolic reaction (Fig. 4C). At least, 35 of 199 enzymes (18%) were found to be involved in multiple metabolic reactions (Supplementary Table S5) mostly related to nucleotide biosynthesis (green circle), fatty acid biosynthesis (purple circle), and amino acid biosynthesis (orange circle). Moreover, some enzymes were identified to play roles in various metabolic reactions across the sub-metabolic pathways (white circle). The complexity introduced by these enzymes is responsible for the connection between the sub-metabolisms of carbon assimilation and the synchronized metabolic behavior^12,43,44^.

**Figure 5:**
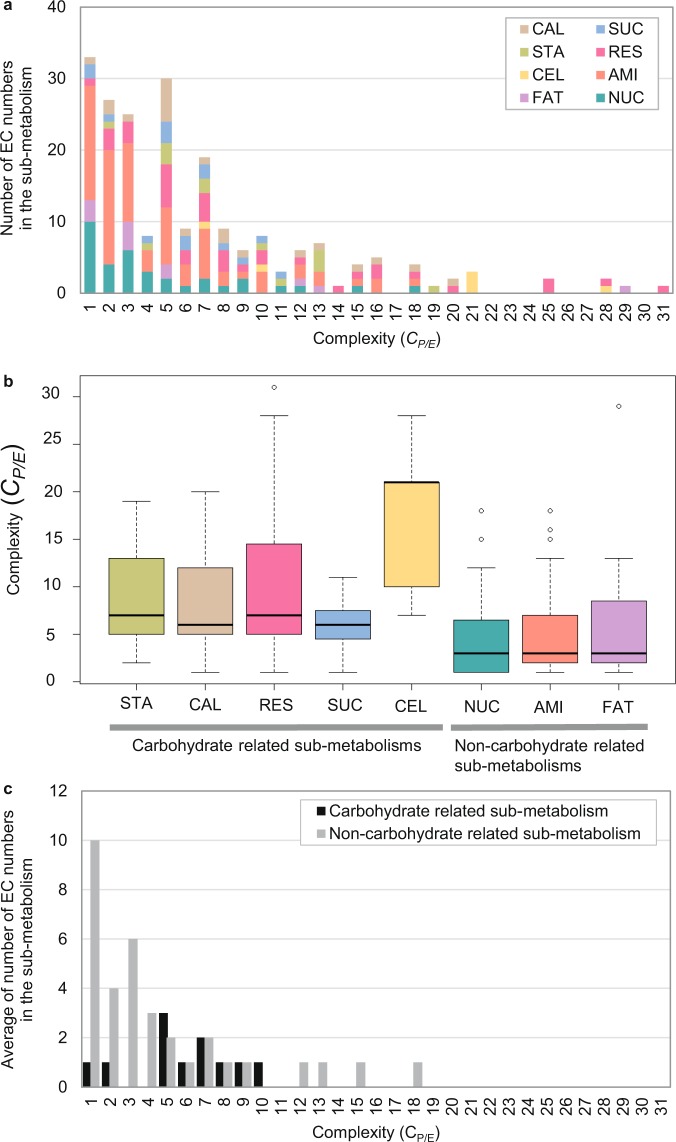
The complexity of the carbon assimilation metabolic pathway of cassava based on association of enzymatic proteins and metabolic enzymes. (**a**) The distribution of proteins annotated to an enzyme (Complexity: *C*_*P/E*_): CAL - Calvin cycle, SUC - sucrose biosynthesis, STA - starch biosynthesis, RES - respiration, AMI - amino acid biosynthesis, CEL - cell wall biosynthesis, FAT - fatty acid biosynthesis, and NUC - nucleotide biosynthesis; (**b**) Box-plot of Complexity (*C*_*P/E*_) determined from each sub-metabolism; (**c**) Complexity (*C*_*P/E*_) of carbohydrate-related sub-metabolisms (black bar: STA, CAL, RES, SUC, and CEL) and non-carbohydrate-related sub-metabolisms (gray bar: NUC, AMI, and FAT) calculated based on the average *C*_*P/E*_ value of enzymes in the pathway.

Furthermore, the metabolic complexity caused by the association of enzymatic proteins, denoted by *C*_*P/E*_ score is shown in Fig. 5A. The number of proteins annotated to an enzyme may suggest the isozyme or protein complex scenario. Fifty-nine percent of enzymes (117/199 EC numbers) in cassava carbon assimilation pathway (MeRecon) were annotated to five proteins or less, while only 18 percent (35/199) contained more than 10 proteins with similar functional annotation. For instance, 28 proteins were annotated to the same enzymatic function as cellulose synthase (EC 2.4.1.12) in the cell wall biosynthesis sub-metabolism. The large cellulose synthase (CesA) gene family is found to catalyze the polysaccharides biosynthesis of cell wall components^45^. We also observed that such complexity was not uniform across all sub-metabolic pathways. For example, the cell wall biosynthesis and respiration pathways contained a larger number of proteins that confer the same enzymatic function compared to others, indicating their superior complexity (Figs 5A,B and S11). Interestingly, protein-to-enzyme association was higher in carbohydrate-related pathways (i.e., starch biosynthesis, Calvin cycle, respiration, sucrose biosynthesis, and cell wall biosynthesis) compared to others sub-metabolisms. On average, more than six proteins were annotated to each enzyme in the carbohydrate-related sub-metabolic pathways, in contrast to three proteins that were annotated to each enzyme in non-carbohydrate-related sub-metabolic pathways, namely nucleotide biosynthesis, amino acid biosynthesis, and fatty acid biosynthesis (Fig. 5B,C). This implies that enzymes (EC number) in carbohydrate-related sub-networks may require more complexity (e.g. protein subunit complex or isozymes) for their function.

### Conservation of cassava carbon assimilation pathway (MeRecon) based upon the model plants

Carbon assimilation is a backbone metabolic process in all living organisms. The pathway is highly conserved across species, especially in those with close evolutionary lineage. Most organisms share overlapping groups of enzymatic reactions, even though the metabolic behavior, i.e. product types and accumulation levels, appear to be diverse. For example, cassava, synthesizes higher amount of starch than other starch crops. To understand this metabolic advantage, this study aimed to decipher the code of metabolic conservation beyond conserved sequences of orthologous enzymatic proteins. The overall structure of the carbon assimilation pathway, the metabolic functions of the sub-metabolic networks, the biochemical properties of enzymes in each sub-metabolism, and the enzymatic function of proteins were analyzed systematically to gain insights into the underlying metabolic processes responsible for the unique starch yield in cassava. The conservation of pathway topology was first investigated through the similarity analysis of the metabolic reactions of the pathway. Then, for each individual sub-metabolism, the functional conservation was examined based on the enzymes (EC numbers) and enzymatic proteins involved in the pathway. Later, the conservation of the biochemical properties was determined for each metabolic reaction based on the variety of proteins involved in the reaction. Finally, for each protein, the enzymatic function was determined based on the evolutionarily preserved sequence.

#### Conservation of MeRecon pathway based on topology

**Figure 6:**
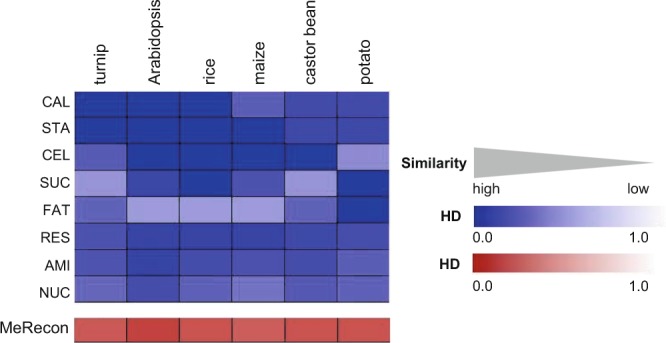
The normalized *Hamming distance* (*HD*) estimates the distance of pathway topology through enzymatic reactions between cassava (MeRecon) and the other six model plants represented as heatmap in blue color. CAL - Calvin cycle, SUC - sucrose biosynthesis, STA - starch biosynthesis, RES - respiration, AMI - amino acid biosynthesis, CEL - cell wall biosynthesis, FAT - fatty acid biosynthesis, and NUC - nucleotide biosynthesis. The heatmap in red represents the *HD* scores that contrasted the topology of the overall carbon assimilation pathway of cassava (MeRecon) with the other plant species.

The metabolic pathway is the series of biochemical reactions driven by enzymes. The combined reactions and enzymes always vary between pathways depending upon the purposes of metabolism. The number of constituent reactions was, thus, considered to reflect the structure of the pathway in this study. To compare the topology of the carbon assimilation pathway of cassava with model plants, normalized *Hamming distance* (*HD*), ranging from 0 (closely related) to 1 (distantly related), was calculated for each sub-metabolism to measure the distance between cassava and each plant template with respect to the co-existing biochemical reactions. The results showed that the overall topology of carbon assimilation pathway in cassava was highly conserved in all six model plants as indicated by very low *HD* scores (Fig. 6). However, difference in pathway topology was observed at sub-metabolism level. The fatty acid biosynthesis pathway in cassava was dissimilar to those in Arabidopsis, rice, maize, which are neither starchy root crops nor of the Euphorbiaceae family. The Calvin cycle and starch biosynthesis sub-metabolism exhibited high topological similarity among all studied plants; however, the Calvin cycle pathway in maize appeared to be the most distantly related cassava. These results may reflect the difference in topology of carbon fixation between the C3 (cassava, Arabidopsis, rice, castor bean, potato, and turnip) and C4 (maize) plant species.

#### Conservation of MeRecon pathway based on metabolic functions of the sub-metabolisms

**Figure 7:**
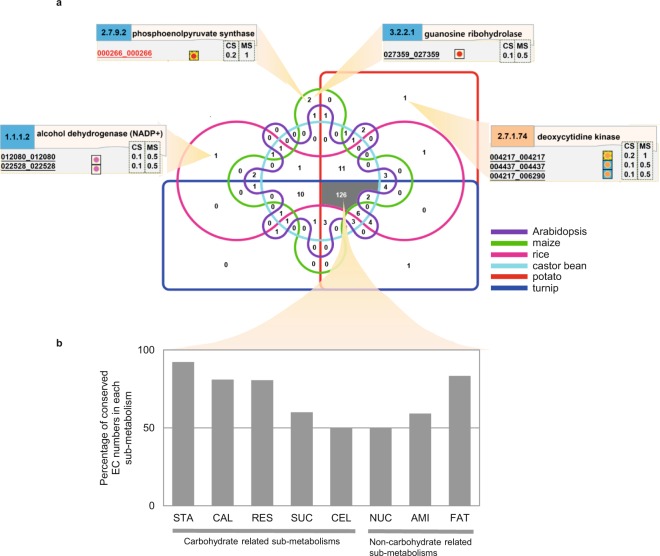
Conservation of metabolic functions of cassava carbon assimilation pathway based on EC numbers (enzymes) with respect to the representative plant species (**a**). The distribution of 126 EC numbers that are conserved across six representative plant species is shown for each sub-metabolism as gray bar (**b**). STA - starch biosynthesis, CAL - Calvin cycle, RES - respiration, SUC - sucrose biosynthesis, CEL - cell wall biosynthesis, NUC - nucleotide biosynthesis, AMI - amino acid biosynthesis, and FAT - fatty acid biosynthesis.

The conservation of metabolic functions was investigated by the similarity of biochemical reactions as well as enzymatic proteins contained in the pathway. The biochemical reactions involved in carbon assimilation in cassava and other plant species were similar (Fig. 7). Sixty-eight percent of the metabolic reactions (175 of 256), corresponding to 126 EC numbers were present in the carbon assimilation pathway of all representative plant species. They were distributed, though not uniformly, across all eight sub-metabolic pathways of cassava carbon assimilation (Fig. 7B). These common metabolic reactions represent a group of primary biochemical reactions that are essential for plants to assimilate carbon to macromolecules. Starch biosynthesis showed the most conserved biochemical reaction constituents of all pathways, whereas cell wall and nucleotide biosynthesis exhibited the least conservation. The common reactions covered at least half of the biochemical reactions in each sub-metabolism, implying that the metabolic function is well conserved in plant species. However, some functional dissimilarity was expressed at sub-metabolism level as a result of some evolutionarily developed reactions. Some metabolic reactions of cassava were found to be conserved in specific template species. This could account for the uniqueness of cassava metabolic pathway. For examples, alcohol dehydrogenase (NADP^+^) (EC 1.1.1.2), phosphoenolpyruvate synthase (EC 2.7.9.2) and guanosine ribohydrolase (EC 3.2.2.1), and deoxycytidine kinase (EC 2.7.1.74) were specifically annotated from rice, maize, and potato, respectively (Fig. 7).

**Figure 8:**
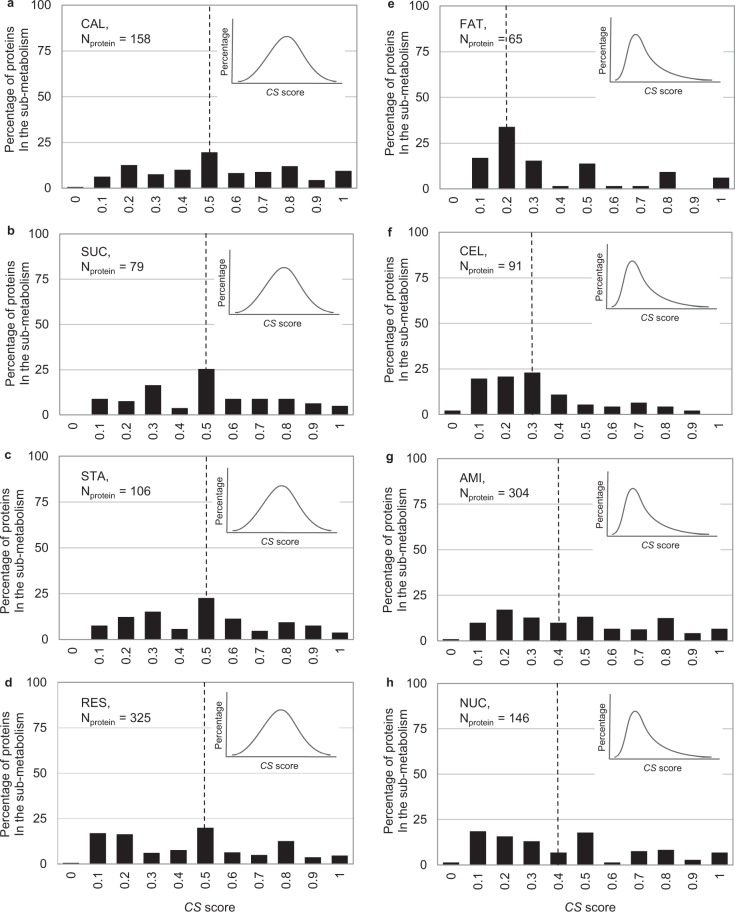
The frequency distribution of conservation score (*CS*) of proteins in sub-metabolisms of cassava carbon assimilation: (**a**) Calvin cycle (**b**) sucrose biosynthesis (**c**) starch biosynthesis (**d**) respiration (**e**) fatty acid biosynthesis (**f**) cell wall biosynthesis (**g**) amino acid biosynthesis, and (**h**) nucleotide biosynthesis. Dotted line represents the median of *CS* score for each the sub-metabolism. The sub-plots on the upper right sides are model distribution curves that estimate symmetric and non-symmetric (right-skewed) distributions.

Similarity in the pathway metabolic function was determined from the constituent biochemical reactions and the conserved proteins. Here, *CS* score^8^ was employed to infer the conservation of enzymatic proteins involved in the pathway. The *CS* score ranged from 0 (no conservation) to 1 (high conservation) and represents the number of template plant species underlying each protein annotation. Figure 8 shows the frequency distribution of *CS* score of 1,046 proteins in the carbon assimilation pathway of cassava. The positively skewed curves of fatty acid biosynthesis, cell wall biosynthesis, amino acid biosynthesis, and nucleotide biosynthesis (Fig. 8E–H) indicate that proteins in these sub-pathways are relatively less conserved in comparison with the rest of carbon assimilation pathways (Fig. 8A–D). Enzymatic proteins in the carbohydrate-related sub-pathways were conserved, albeit moderately as indicated by the median *CS*-score of about 0.5. The results suggest variation of metabolic function at the sub-metabolism level. Next, we investigated whether variations in constituent enzymatic proteins affected the metabolic reaction.

#### Conservation of MeRecon pathway based on biochemical properties of enzymes in each sub-metabolism

**Figure 9:**
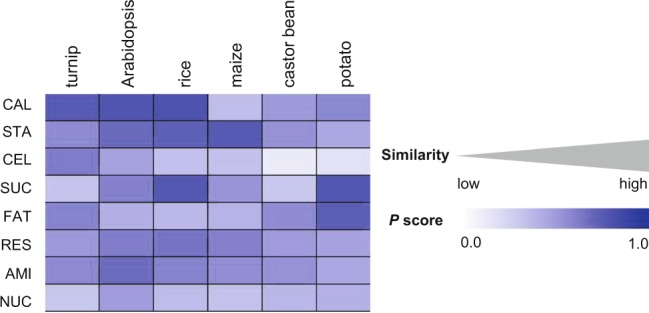
The *P* score of each sub-metabolism in cassava carbon assimilation pathway (MeRecon) based on annotated proteins within an EC number (enzyme) of all enzymes from template plants. CAL - Calvin cycle, STA - starch biosynthesis, CEL - cell wall biosynthesis, SUC - sucrose biosynthesis, FAT - fatty acid biosynthesis, RES - respiration, AMI - amino acid biosynthesis, and NUC - nucleotide biosynthesis.

In this study, the complexity (*C*_*P/E*_ score) of each metabolic reaction was used to represent the biochemical property, and its conservation in cassava and representative plant species was examined based upon the *P* score, which ranges from 0 (dissimilar) to 1 (identical) and indicates the tendency that two contrasting metabolic reactions possess similar level of complexity (*C*_*P/E*_). The complexity of reactions in MeRecon differed from those in the representative plant species (Fig. 9). The pattern of *P* score indicated less conserved biochemical property of enzymes at sub-metabolism level, corresponding to the results of conservation in the pathway topology and metabolic function. This difference is more pronounced in metabolic reaction as observed between cassava and the model plant species. For example, the metabolic function and topology of the cell wall biosynthesis sub-metabolism is conserved across template plants (Fig. 6), while the biochemical properties of enzymes vary (low *P* score in Fig. 9). The results indicate that complete metabolic behavior could not be inferred based only on pathway topology conservation. The distinct complexity of constituent reactions might explain the variations in metabolic behavior, despite the highly conserved carbon assimilation pathway.

#### Conservation of MeRecon pathway based on enzymatic function of proteins

The previous sections highlighted the conservation in the topology, metabolic function, and biochemical properties of reactions in the carbon assimilation network of cassava and revealed variations in enzymatic reactions. Here, we investigated the conservation of enzymatic protein functions based on the conserved amino acid sequences. Utilization of metabolic substrates by the action of enzymatic proteins may be affected by variation in amino acid sequences. Two proteins, namely, phosphoenolpyruvate synthase (EC 2.7.9.2; PEPS) and aldehyde dehydrogenase (NAD^+^) (EC 1.2.1.3; ALDH) were selected to demonstrate the conservation and possible variations in enzymatic functions. A reaction driven by PEPS (R00199) was reconstructed in MeRecon using the PEPS cassava protein, MeRecon: 000266_000266, annotated from maize, with high sequence similarity. The ALDH is a large class of enzyme relevant to numerous proteins.

**Figure 10:**
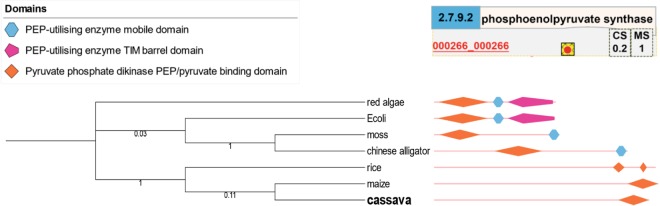
The phylogenetic analysis of the cassava protein — 000266_000266 in template species, and visualization of phosphoenolpyruvate synthase (EC 2.7.9.2) in SmartDraw. Evolution of the cassava protein sequence — 000266_000266 was analyzed by maximum likelihood (ML) tree. The ML tree was reconstructed with Jones-Taylor-Thornton (JTT) amino acid substitution model. Bootstrap values expressed as percentages of 100 replications are shown near the branch points. Phosphoenolpyruvate synthase (EC 2.7.9.2) was annotated from maize (red circle) and visualized in SmartDraw platform (Top right). The domain families are marked with different shapes and colors.

PEPS catalyzes the conversion of pyruvate to phosphoenolpyruvate. The PEPS protein (MeRecon: 000266_000266; Fig. 10) was annotated to the carbon assimilation pathway of cassava from its orthologue in maize, but not rice despite its occurrence in the latter due failure to meet the set criteria (% identity = 59.28). In contrast, the PEPS protein (000266_000266) was highly similar to the orthologue in maize (*MS* score = 1; Fig. 10) in terms of amino acid sequence. Its existence in cassava metabolism was also supported by the cloned sequence obtained from the GenBank database and the expression data^33^. To investigate the conservation of PEPS enzymatic function, the protein domain of the 000266_000266 protein was analyzed and compared with its orthologues in other species, including maize and rice (representatives of plants), moss (a representative of primitive plants), Chinese alligator (a representative of animals), *Escherichia coli* (a representative of bacteria), and red algae (a representative of algae). The 000266_000266 protein contained the PEP/pyruvate binding domain, a typical domain found in PEPS proteins of all species (Fig. 10). The results also highlighted the evolution of amino acid sequence and the structure of the protein domain in 000266_000266 protein of cassava. PEPS protein evolved towards its orthologues in higher plant species, especially maize. Evolution of amino acid sequence could affect the enzymatic function of proteins, and it has been hypothesized to contribute to the distinct behavior of conserved metabolic pathways. Like pyruvate and phosphate dikinase (PPDK), cassava PEPS (000266_000266) is a member of the PEP-utilizing enzyme family that catalyzes the conversion of pyruvate to phosphoenolpyruvate (PEP), and *vice versa*^46^. PEPS was reported to have similar structure and function as PPDK, an important photosynthetic enzyme found in C4 plants like maize^46,47^. PEP is utilized for carboxylation (CO_2_ fixation) by phosphoenolpyruvate carboxylase (PEPC)^48^. The activity of PEPC is much higher in cassava than in typical C3-plants^49^. In stress environment, such as high temperature and soil water stress, PEPC of cassava has greater activity and affinity to CO_2_ than ribulose-1,5-bisphophate carboxylase/oxygenase (Rubisco)^50^, which might explain the high photosynthetic capacity of cassava over C3-plants^50^ leading to its unique carbon assimilation, although it lacks the C4-Kranz anatomy^51^.

**Figure 11:**
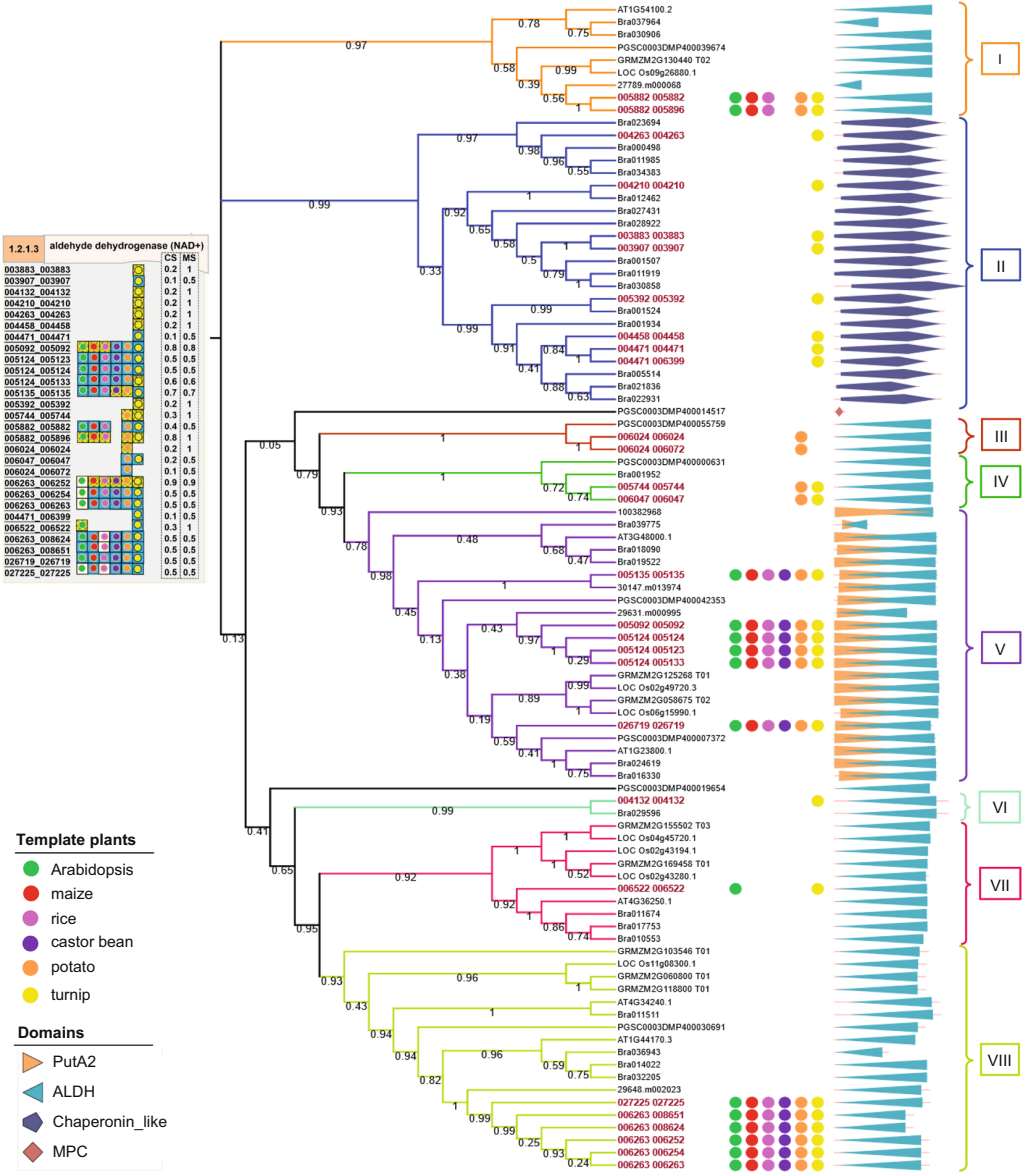
The maximum likelihood (ML) tree and domain analysis among 28 amino acid sequences of cassava (red) annotated to aldehyde dehydrogenase (NAD^+^) (EC 1.2.1.3; ALDH) and amino acid sequences of template plants (black). The ML tree was reconstructed with Jones-Taylor-Thornton (JTT) amino acid substitution model. Bootstrap values were also applied with 100 replicates represented as percentage on the tree branches. The shapes and colors represent the domain families: Aldehyde-alcohol dehydrogenase (PutA2), Aldehyde dehydrogenase (ALDH), chaperonin like, and the uncharacterized protein family (MPC). The representative model plant species, marked as colored circles, were used to annotate each cassava protein. All of the ALDH proteins in cassava (MeRecon) was also visualized in SmartDraw platform (Top left).

Aldehyde dehydrogenase (NAD+) (EC 1.2.1.3; ALDH) is responsible for converting acetaldehyde to acetate in the respiration sub-metabolism. ALDH belongs to a large protein ALDH superfamily that catalyze the detoxification of board aldehydes to their corresponding carboxylic acids through oxidation^52^. The ALDH superfamily in plants comprises 13 distinct families^53^. Fourteen genes encoding proteins in Arabidopsis were found to be members of nine ALDH families^54^. ALDH plays a role in various metabolic processes, including carnitine biosynthesis, glycolysis, gluconeogenesis and amino acid metabolism^53^; and is proposed as one of the important enzymes that promote plant growth under abiotic stress conditions, e.g. drought stress, due to its high expression in stress-response pathways related to plant resistance^53,55^. Conservation of the 28 ALDH protein sequences (19 genes) in cassava (MeRecon): 003883_003883, 003907_003907, 004132_004132, 004210_004210, 004263_004263, 004458_004458, 004471_004471, 004471_006399, 005092_005092, 005124_005123, 005124_005124, 005124_005133, 005135_005135, 005392_005392, 005744_005744, 005882_005882, 005882_005896, 006024_006024, 006024_006072, 006047_006047, 006263_006252, 006263_006254, 006263_006263, 006263_008624, 006263_008651, 006522_006522, 026719_026719, and 027225_027225) was studied with respect to 65 orthologous proteins in template plant species (Fig. 11). The ALDH enzyme was highly conserved among the templates, but was diverged among groups of corresponding proteins. Although the important ALDH domain (marked as blue) was conserved in almost all proteins, variations in amino acid sequences were observed. The ALDH enzymatic proteins of groups I, V, and VIII (005882_005882, 005882_005896, 026719_026719, 005135_005135, 005092_005092, 005124_005133, 005124_005123, 005124_005124, 027225_027225, 006263_006252, 006263_006254, 006263_006263, 006263_008624, and 006263_008651) occurred in most of the templates, while members of groups II, III, IV, VI, and VII were conserved in a narrow group of template plants, suggesting that they evolved towards a particular enzyme function. Furthermore, the group II ALDH enzymatic proteins had distinct protein domain and were conserved only in turnip, a representative of starchy root crops. Even though the overall pathway topology and metabolic function of the sub-pathways seemed identical across the representative model plants, variations in the protein sequences that were annotated to particular enzyme could impact enzymatic functions thereby affecting the metabolic processes.

## Conclusions

The carbon assimilation pathway of cassava (MeRecon) was reconstructed and subsequently investigated through comparative network analysis to identify conserved and diverged metabolic characteristics of cassava that might relate to the distinct metabolic phenotype of cassava in terms of starch production. First, MeRecon pathway was reconstructed using comparative genomic approach based upon annotated cassava enzymatic proteins from six plant templates. Reaction gaps were filled using cassava enzymatic proteins obtained from the CassavaCyc database. The MeRecon pathway consists of 199 EC numbers (enzymes), 259 reactions, 259 metabolites, 870 genes, and 1,052 proteins. More than 95 annotated enzymatic proteins were validated using either the six cassava transcriptome datasets or the cloned sequences of cassava that were obtained from GenBank database or both when available. Based on the complexity analysis (*C*_*P/E*_) of MeRecon pathway, the carbohydrate-related sub-metabolisms may require higher enzymatic protein complexity (i.e., protein subunit complex or isozymes) for their functions compared to non-carbohydrate sub-metabolisms. In addition, the MeRecon pathway was compared to the six model plants with respect to pathway topology, metabolic function of sub-metabolisms, biochemical properties of enzymes in each sub-metabolism, and enzymatic function of proteins. Although the topology and metabolic function were highly conserved across templates, different enzyme properties and enzymatic function of proteins were identified which might affect the synthesis of metabolic compounds and the global metabolic behavior.

## Electronic supplementary material

Supplementary Information

Supplementary Table S3

Supplementary Table S4
