# Supplementary material for: Unlocking conserved and diverged metabolic characteristics in cassava carbon assimilation via comparative genomics approach
Source: Sci Rep. 2018 Nov 9;8:16593. doi: 10.1038/s41598-018-34730-y (PMC6226483; doi:10.1038/s41598-018-34730-y)
Supplement: Supplementary file 1 — Supplementary Information [file 41598_2018_34730_MOESM1_ESM.pdf]

Unlocking conserved and diverged metabolic characteristics in cassava carbon assimilation via comparative genomics approach

Wanatsanan Siriwat[1], Saowalak Kalapanulak[1,2], Malinee Suksangpanomrung[3], and Treenut Saithong[1,2,*]

[1]Systems Biology and Bioinformatics Research Laboratory, Pilot Plant Development and Training Institute, King Mongkut's University of Technology Thonburi, Bang Khun Thian, Bangkok, 10150, Thailand
[2]Bioinformatics and Systems Biology Program, School of Bioresources and Technology, King Mongkut's University of Technology Thonburi, Bang Khun Thian, Bangkok, 10150, Thailand
[3]National Center for Genetic Engineering and Biotechnology, Pathum Thani, 12120, Thailand

[*]E-mail: treenut.sai@kmutt.ac.th

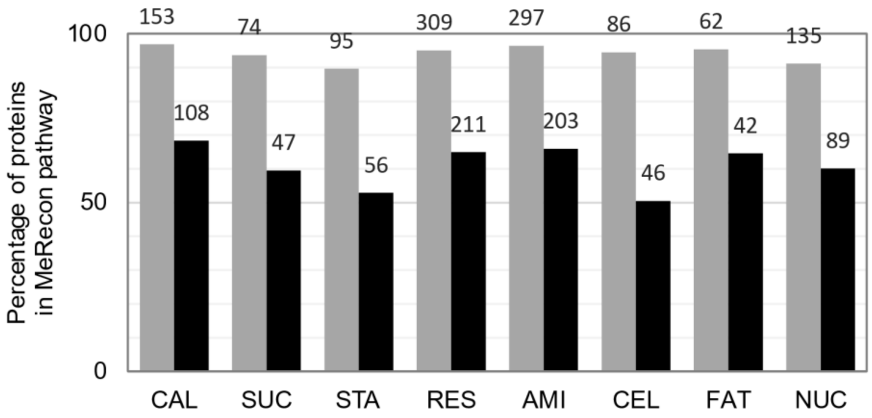

Figure S1. The percentage of proteins in cassava carbon assimilation pathway (MeRecon) that were supported by six transcriptome datasets on: leaf and stem[30], root development[30-32], cold stress[33], drought stress[34], and fungal infection[35]. Gray bars show proteins supported by at least one transcriptome dataset, while black bars show proteins supported by at least two transcriptome datasets. The numbers at the top of each bar show the number of proteins. CAL - Calvin cycle, SUC - sucrose biosynthesis, STA - starch biosynthesis, RES - respiration, AMI - amino acid biosynthesis, CEL - cell wall biosynthesis, FAT - fatty acid biosynthesis, and NUC - nucleotide biosynthesis.

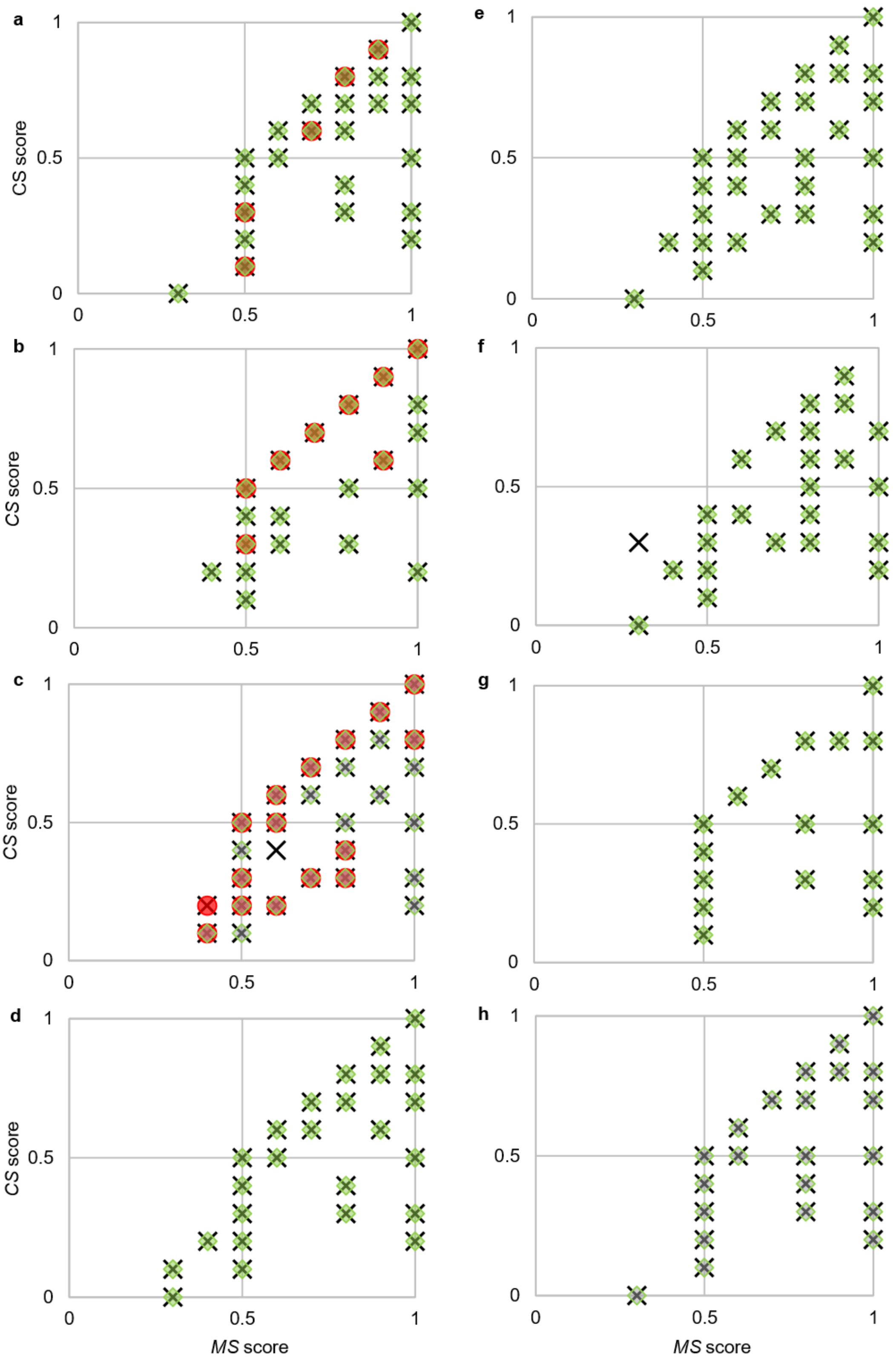

Figure S2. The *MS-CS* plot of annotated proteins in MeRecon pathway: (**a**) Calvin cycle, (**b**) sucrose biosynthesis, (**c**) starch biosynthesis, (**d**) respiration, (**e**) amino acid biosynthesis, (**f**) cell wall biosynthesis, (**g**) fatty acid biosynthesis, and (**h**) nucleotide biosynthesis sub-metabolisms.

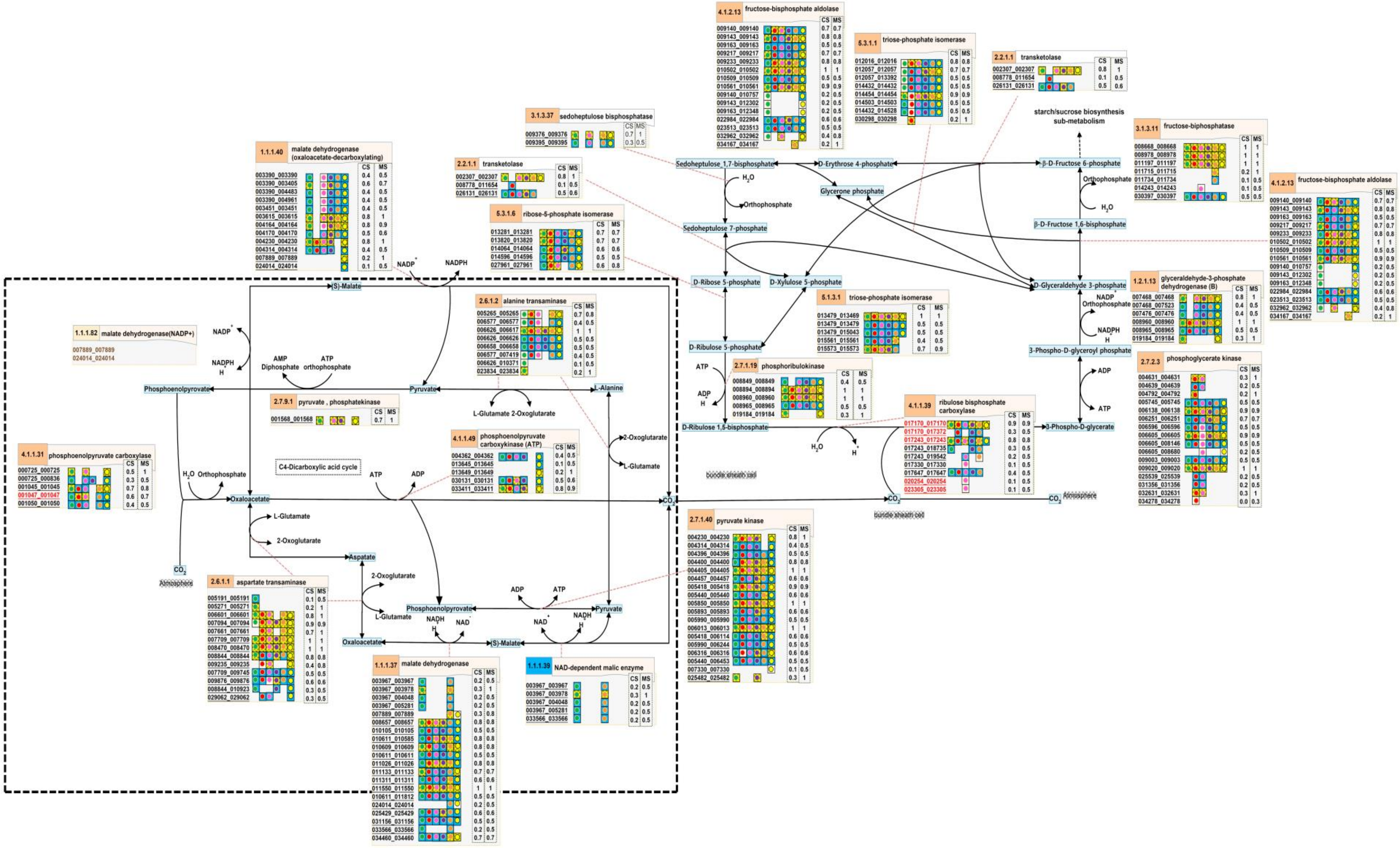

Figure S3. Calvin cycle sub-metabolism in MeRecon visualized by SmartDraw. (High quality figure can be downloaded at http://bml.sbi.kmutt.ac.th/MeRecon)

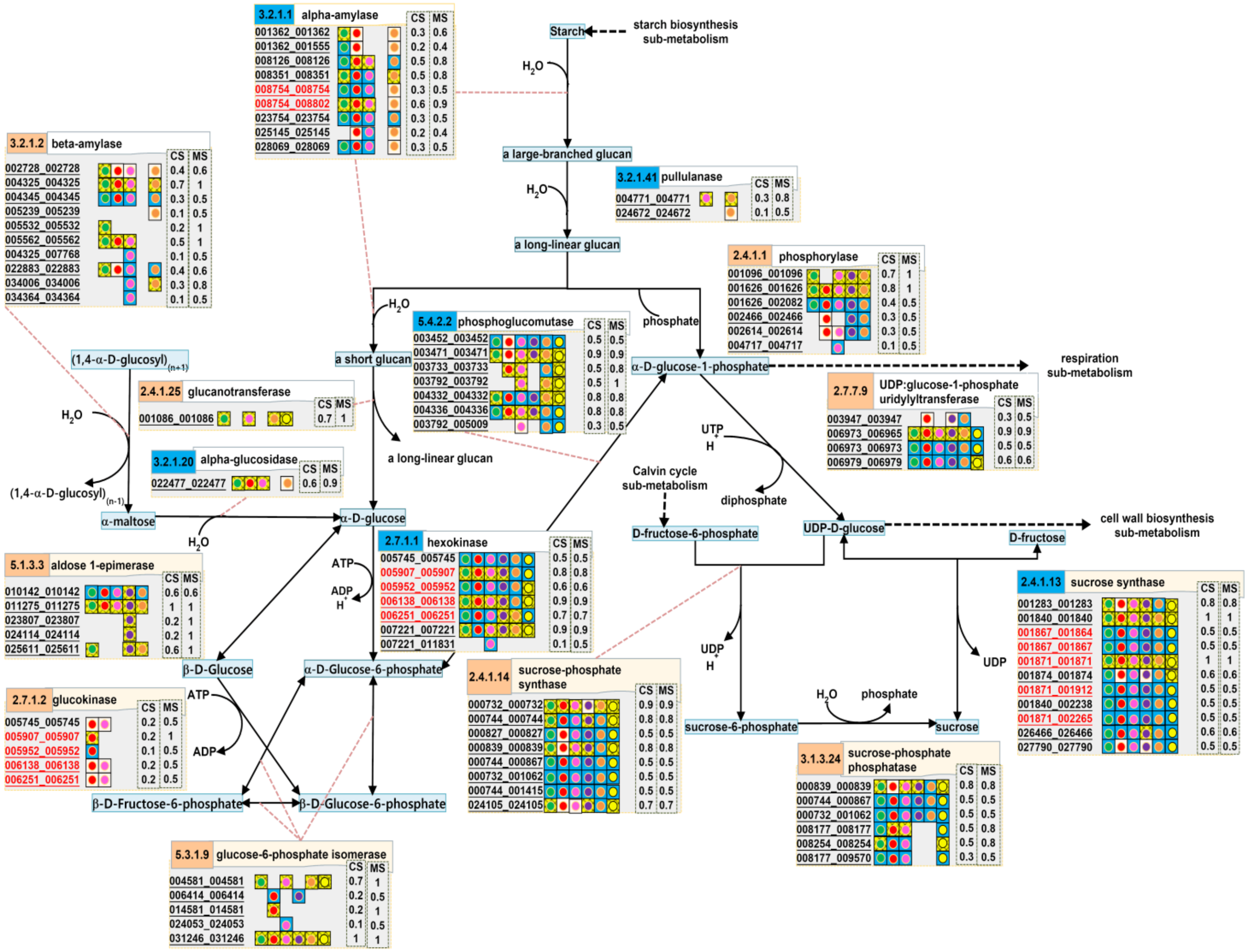

Figure S4. Sucrose biosynthesis sub-metabolism in MeRecon visualized by SmartDraw. (High quality figure can be downloaded at http://bml.sbi.kmutt.ac.th/MeRecon)

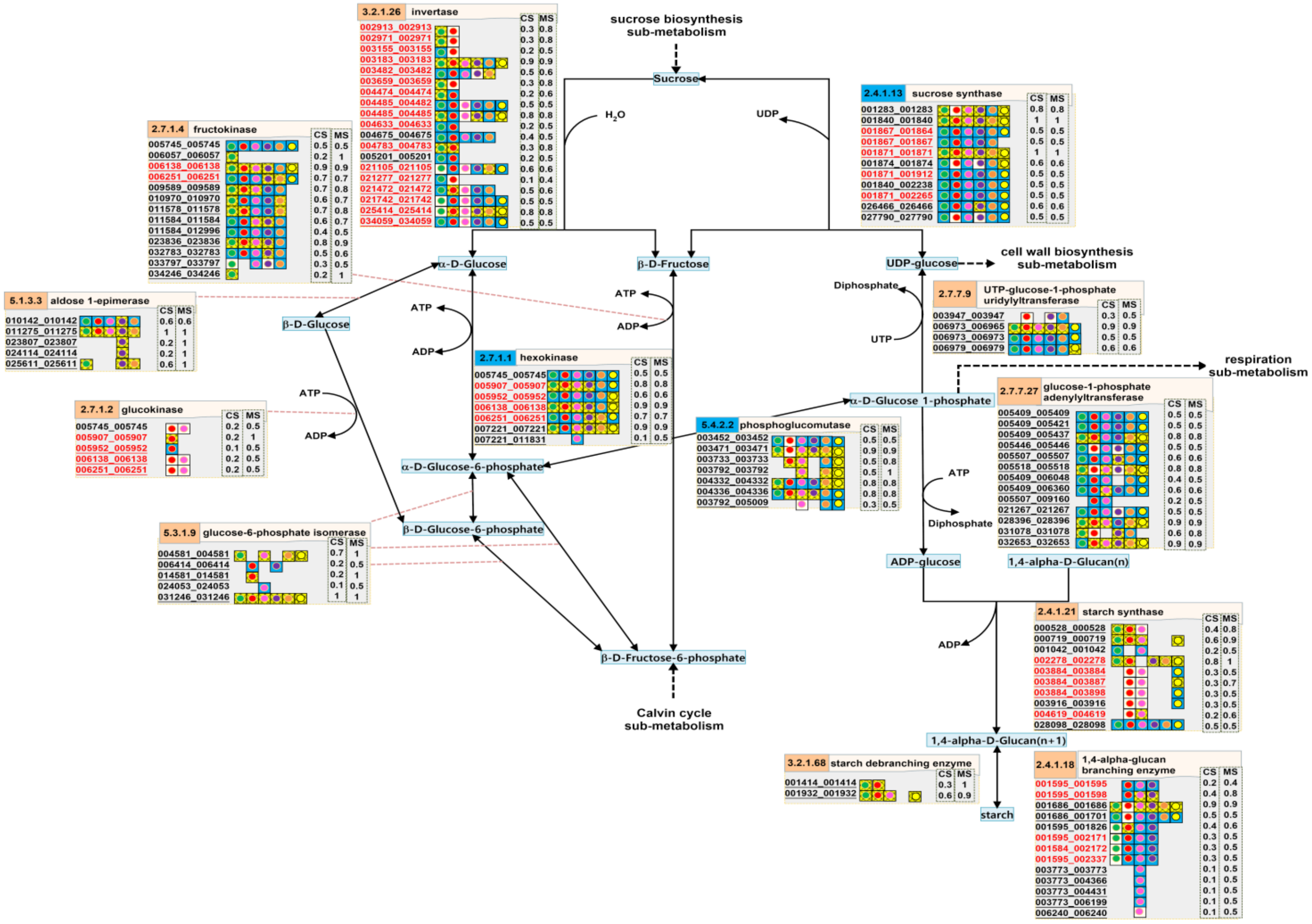

Figure S5. Starch biosynthesis sub-metabolism in MeRecon visualized by SmartDraw. (High quality figure can be downloaded at http://bml.sbi.kmutt.ac.th/MeRecon)

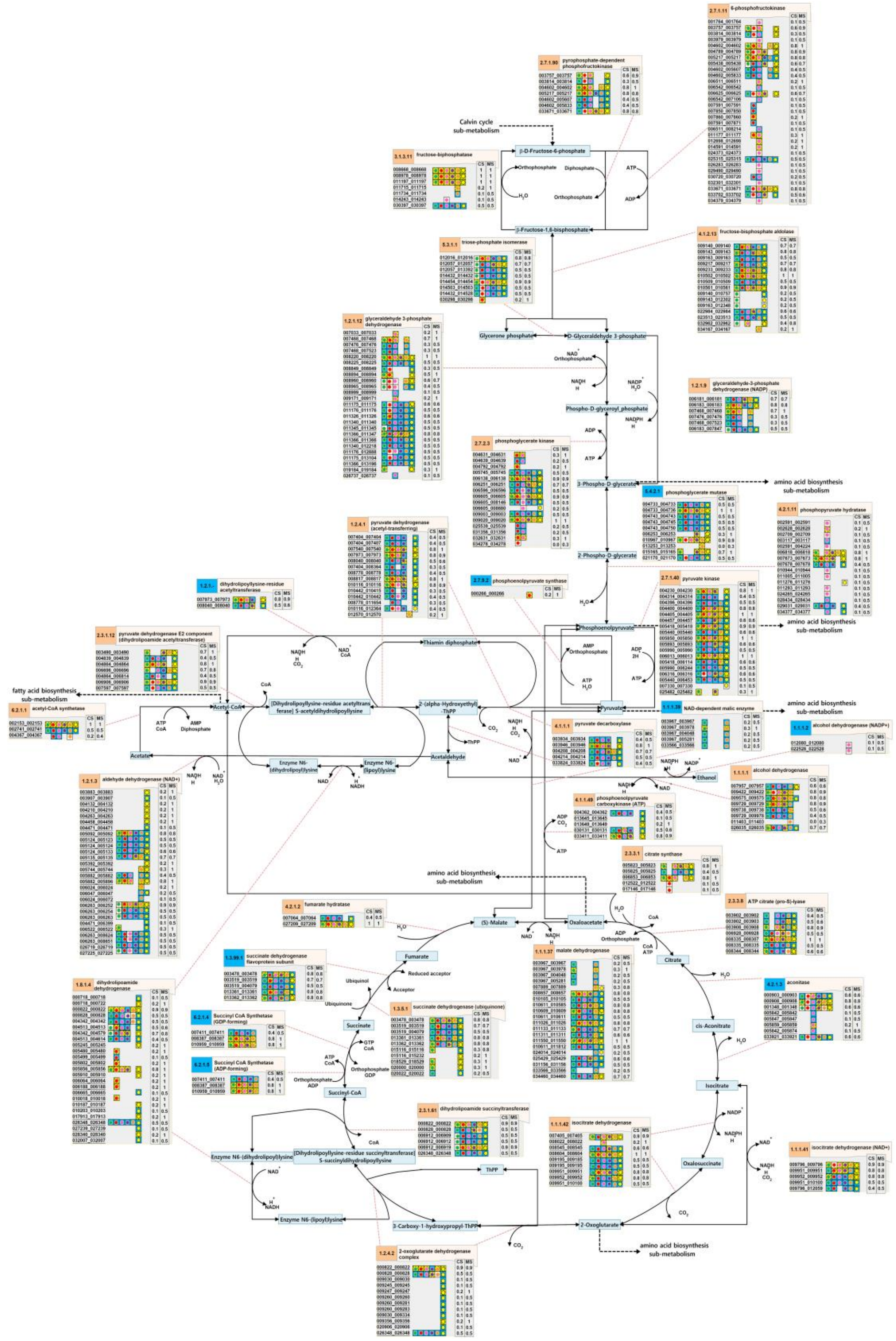

Figure S6. Respiration sub-metabolism in MeRecon visualized by SmartDraw. (High quality figure can be downloaded at http://bml.sbi.kmutt.ac.th/MeRecon)

a

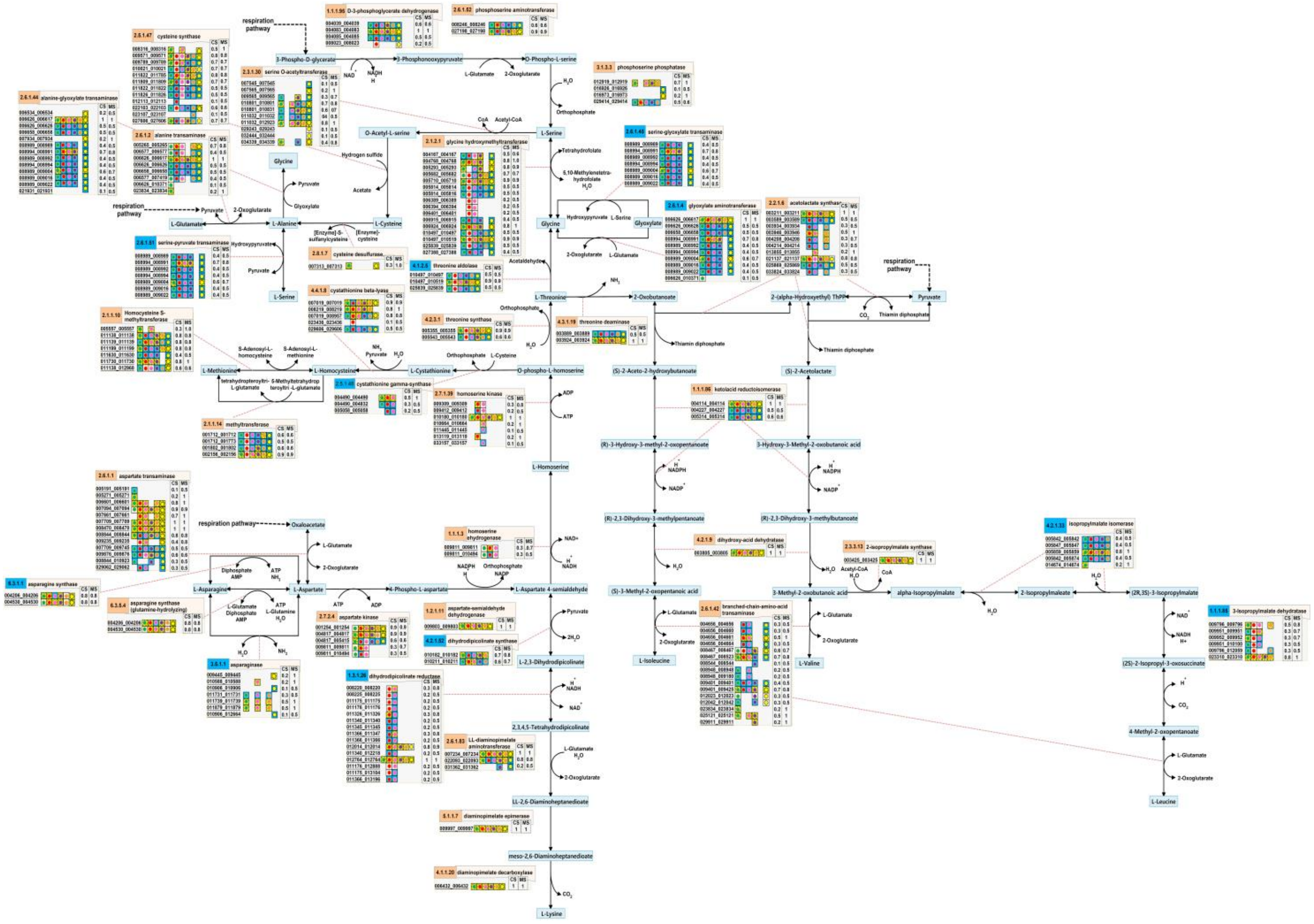

Figure S7. Amino acid biosynthesis sub-metabolism in MeRecon visualized by SmartDraw: (**a**) Serine, Glycine, Threonine, Cysteine, Methionine, Aspartate, Asparagine, Isoleucine, Valine, Leucine, Lysine, and Alanine, (**b**) Glutamate, Glutamine, Proline, and Arginine, (**c**) Histidine, and (**d**) Phenylalanine, Tyrosine, and Tryptophan. (High quality figure can be downloaded at http://bml.sbi.kmutt.ac.th/MeRecon)

b

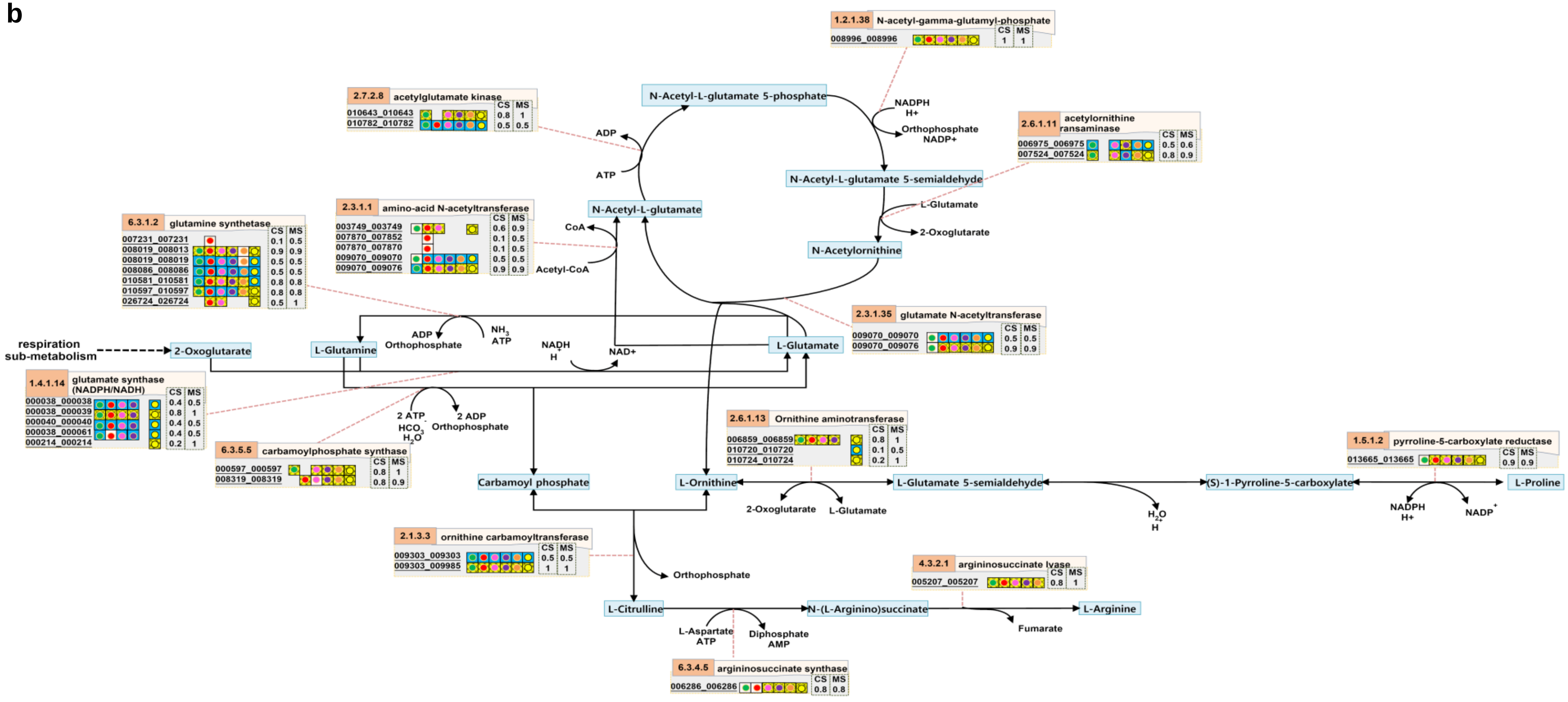

Figure S7. Amino acid biosynthesis sub-metabolism in MeRecon visualized by SmartDraw: (**a**) Serine, Glycine, Threonine, Cysteine, Methionine, Aspartate, Asparagine, Isoleucine, Valine, Leucine, Lysine, and Alanine, (**b**) Glutamate, Glutamine, Proline, and Arginine, (**c**) Histidine, and (**d**) Phenylalanine, Tyrosine, and Tryptophan. (High quality figure can be downloaded at http://bml.sbi.kmutt.ac.th/MeRecon)

c

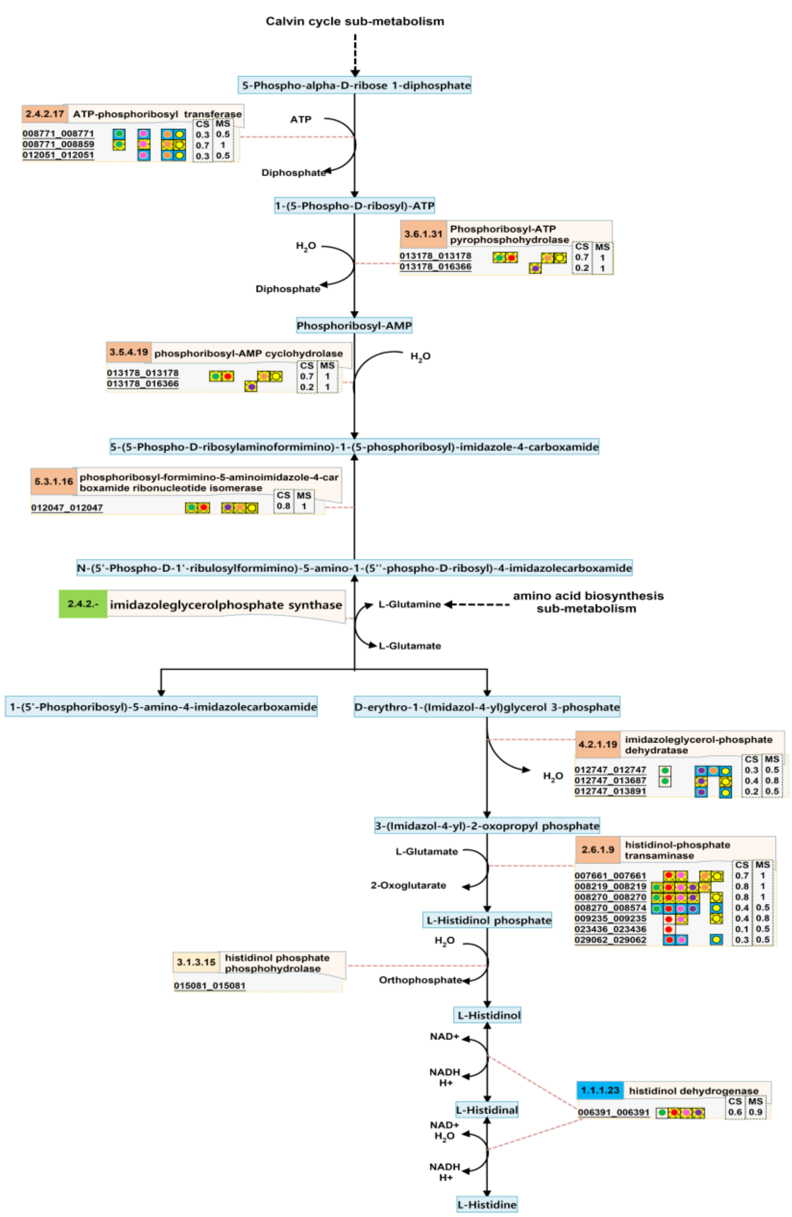

Figure S7. Amino acid biosynthesis sub-metabolism in MeRecon visualized by SmartDraw: (**a**) Serine, Glycine, Threonine, Cysteine, Methionine, Aspartate, Asparagine, Isoleucine, Valine, Leucine, Lysine, and Alanine, (**b**) Glutamate, Glutamine, Proline, and Arginine, (**c**) Histidine, and (**d**) Phenylalanine, Tyrosine, and Tryptophan. (High quality figure can be downloaded at http://bml.sbi.kmutt.ac.th/MeRecon)

d

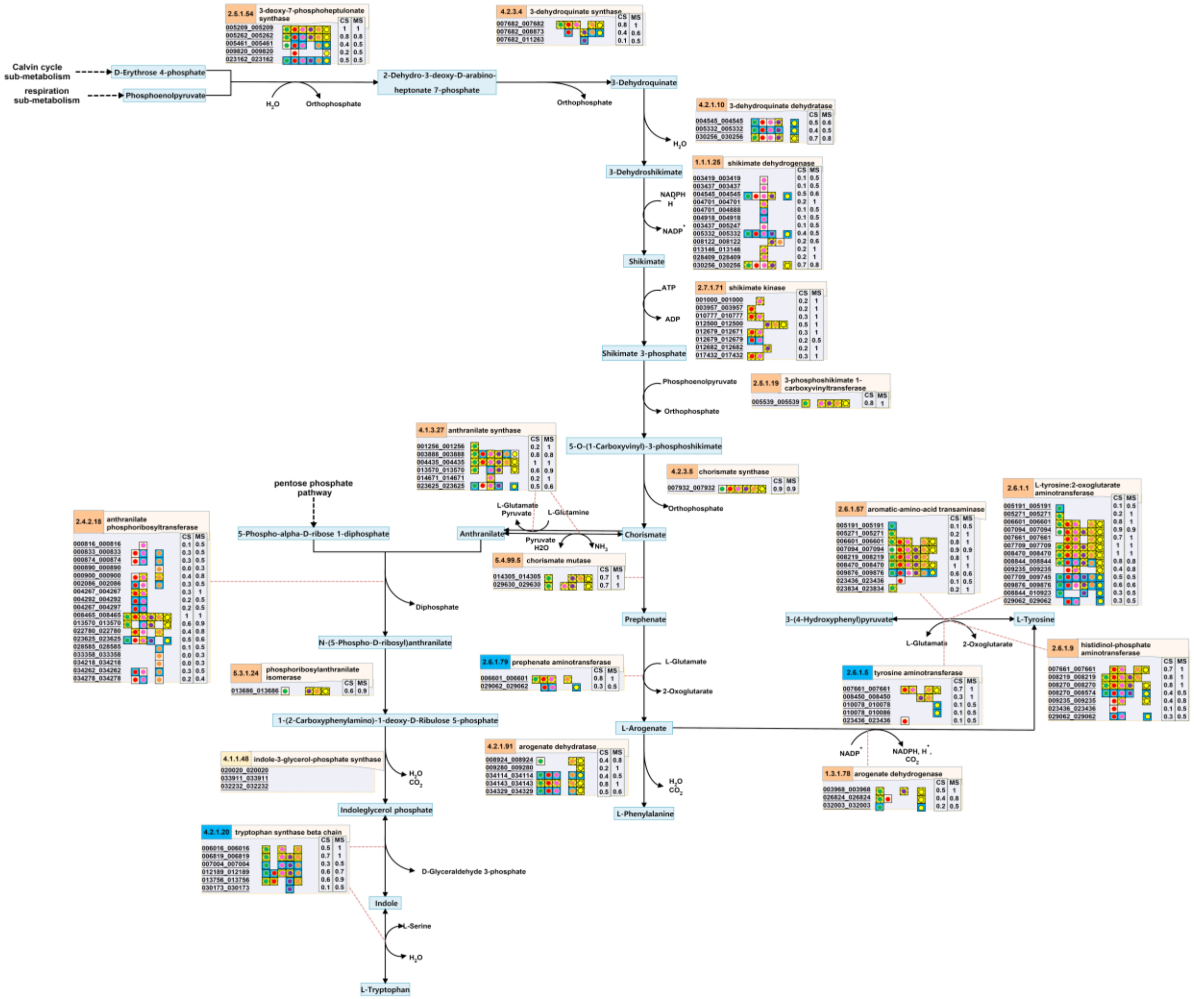

Figure S7. Amino acid biosynthesis sub-metabolism in MeRecon visualized by SmartDraw: (**a**) Serine, Glycine, Threonine, Cysteine, Methionine, Aspartate, Asparagine, Isoleucine, Valine, Leucine, Lysine, and Alanine, (**b**) Glutamate, Glutamine, Proline, and Arginine, (**c**) Histidine, and (**d**) Phenylalanine, Tyrosine, and Tryptophan. (High quality figure can be downloaded at http://bml.sbi.kmutt.ac.th/MeRecon)

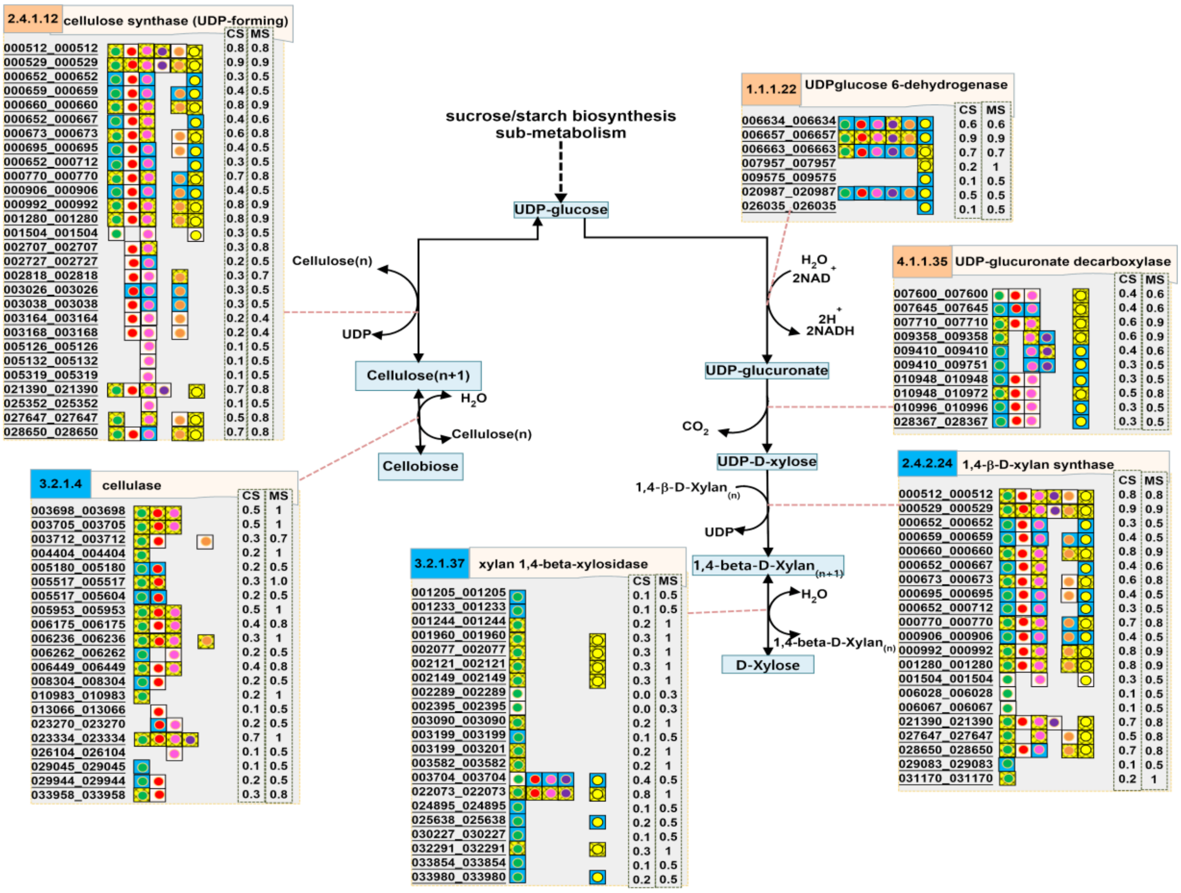

Figure S8. Cell wall biosynthesis sub-metabolism in MeRecon visualized by SmartDraw. (High quality figure can be downloaded at http://bml.sbi.kmutt.ac.th/ MeRecon)

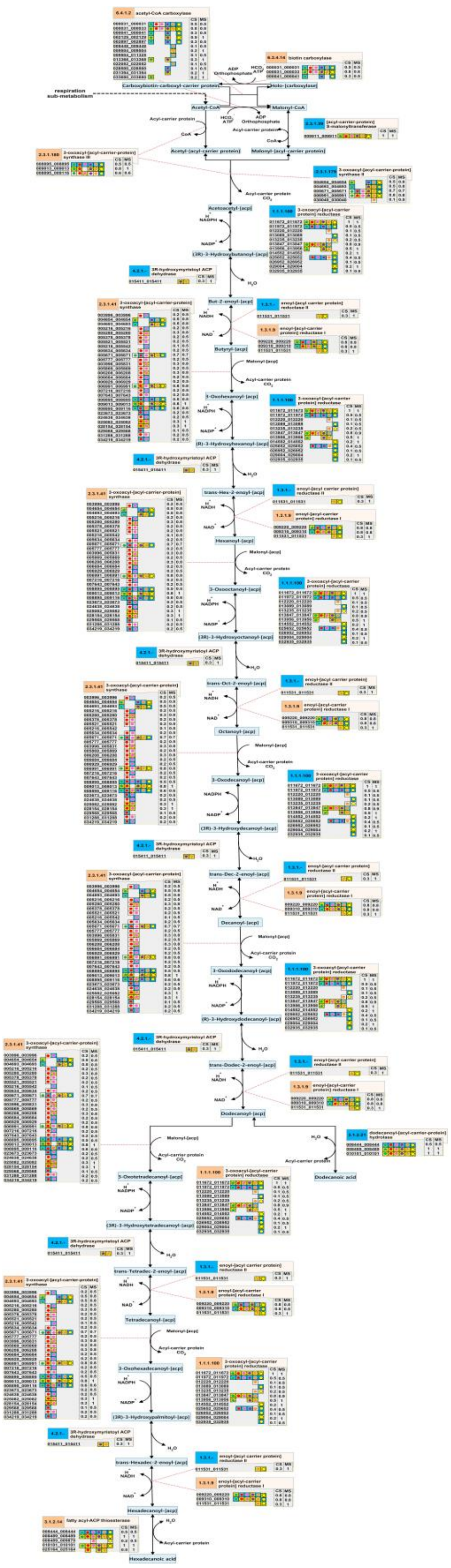

Figure S9. Fatty acid biosynthesis sub-metabolism in MeRecon visualized by SmartDraw. (High quality figure can be downloaded at http://bml.sbi.kmutt.ac.th/MeRecon)

a

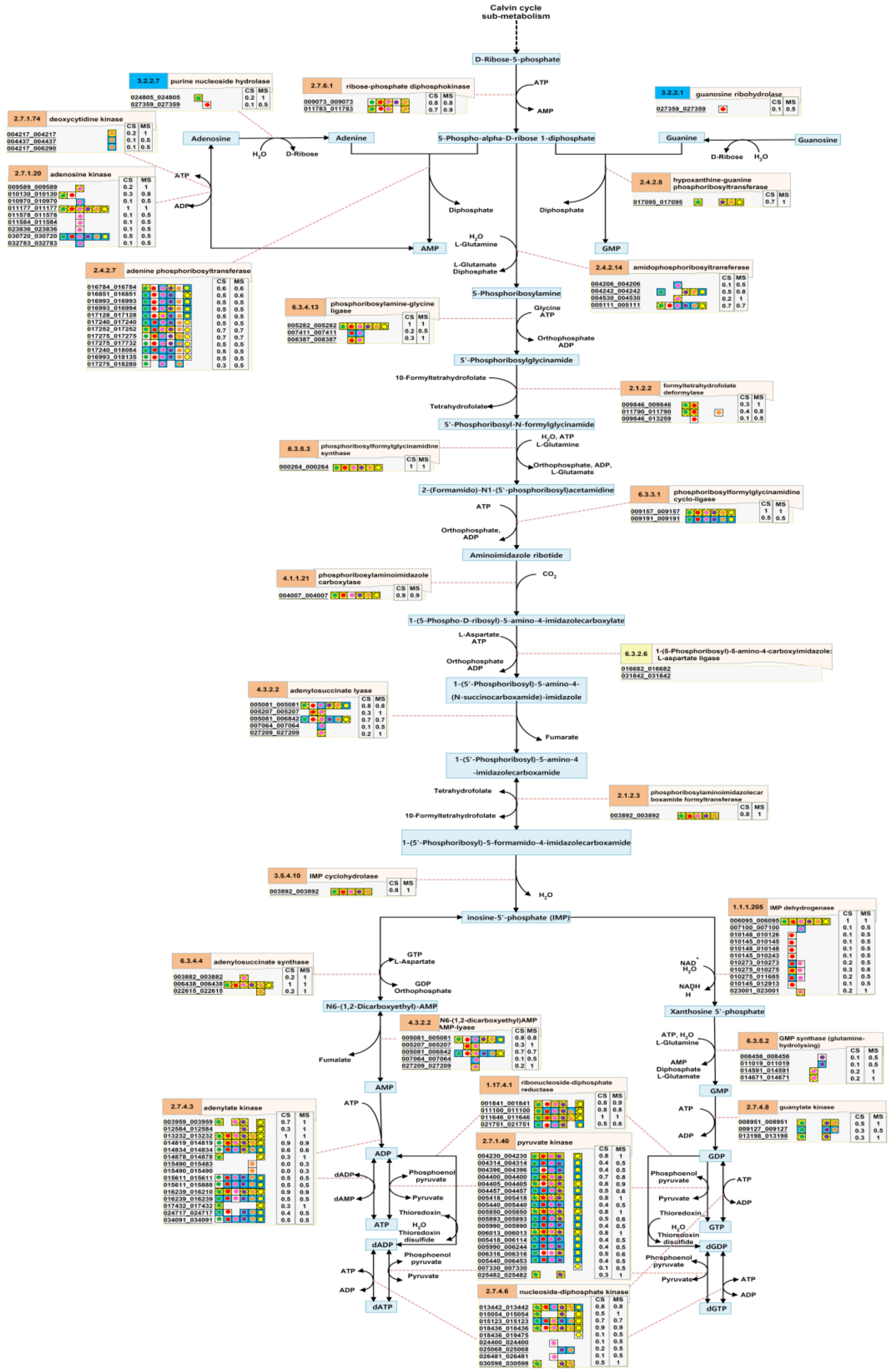

Figure S10. Nucleotide biosynthesis sub-metabolism in MeRecon visualized by SmartDraw: (**a**) purine and (**b**) pyrimidine. (High quality figure can be downloaded at http://bml.sbi.kmutt. ac.th/MeRecon)

b

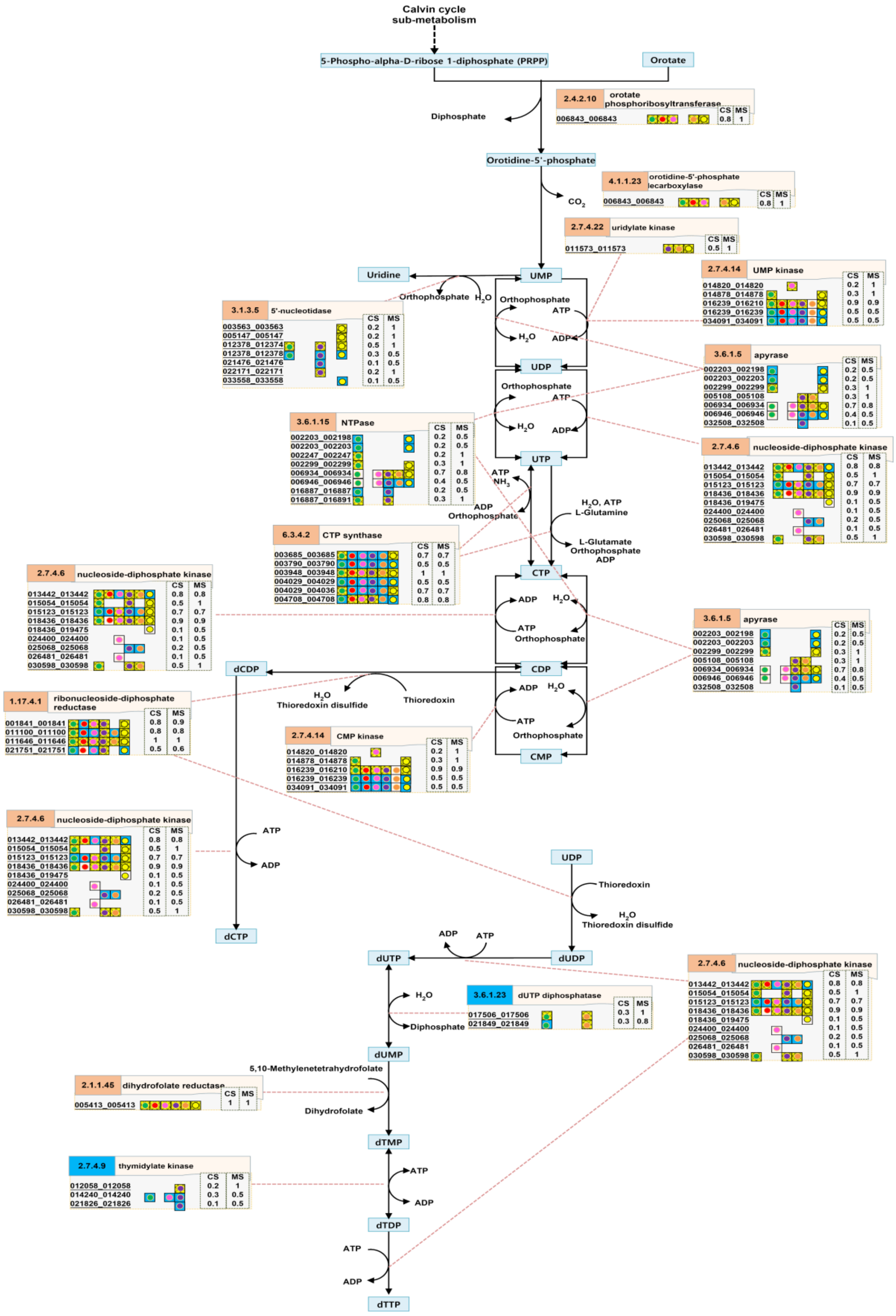

Figure S10. Nucleotide biosynthesis sub-metabolism in MeRecon visualized by SmartDraw: (**a**) purine and (**b**) pyrimidine. (High quality figure can be downloaded at http://bml.sbi.kmutt. ac.th/MeRecon)

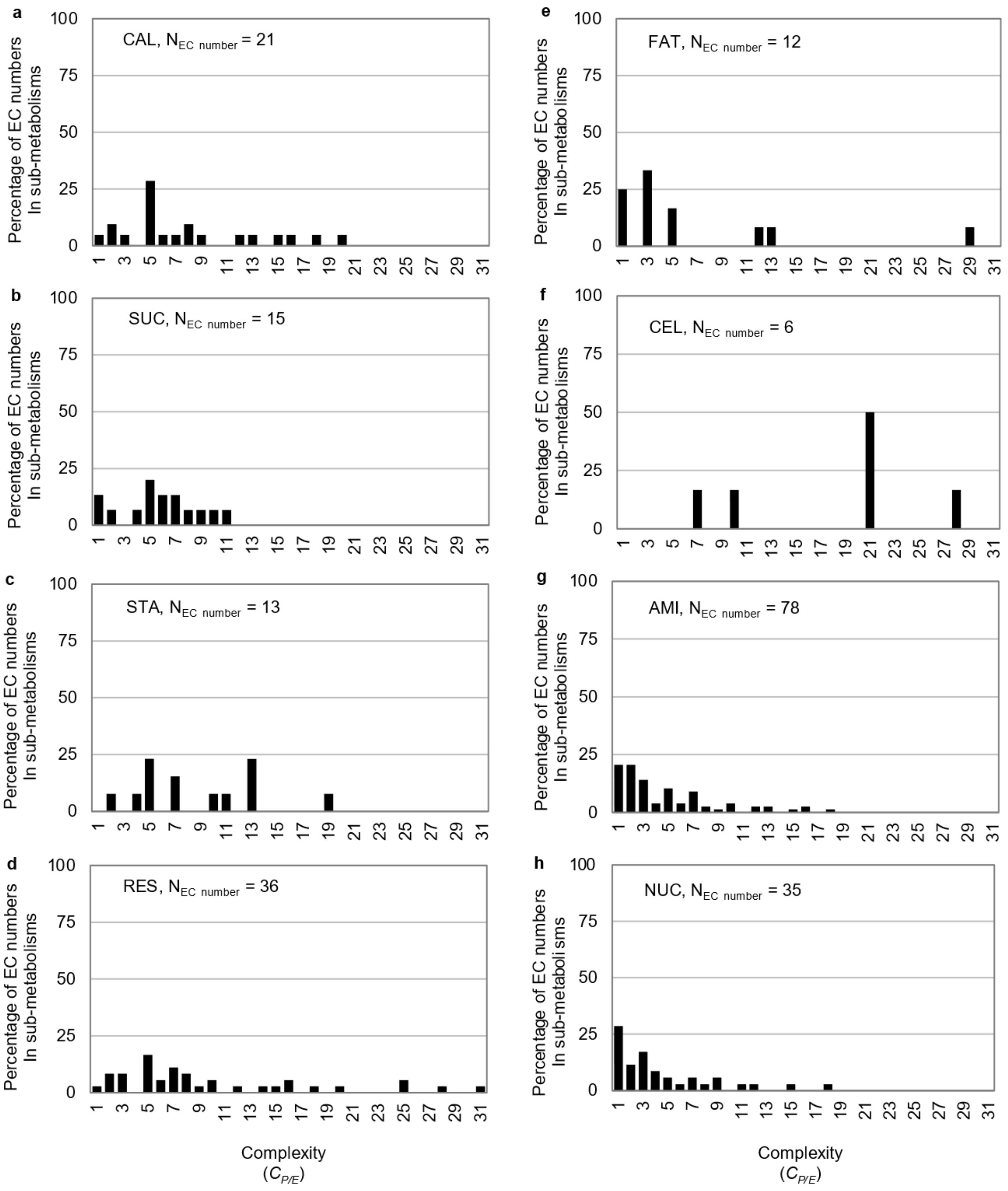

Figure S11. The distribution of proteins annotated to enzymes (Complexity: $C_{P/E}$) in the carbon assimilation metabolic pathway of cassava: (**a**) Calvin cycle, (**b**) sucrose biosynthesis, (**c**) starch biosynthesis, (**d**) respiration, (**e**) fatty acid biosynthesis, (**f**) cell wall biosynthesis, (**g**) amino acid biosynthesis, and (**h**) nucleotide biosynthesis.

Table S1. The databases from which plant genomic data were retrieved.

Plants	Genome databases
**Arabidopsis** (*Arabidopsis thaliana*)	The Arabidopsis Information Resource (TAIR) v10
**Maize** (*Zea mays*)	Phytozome v7.0
**Rice** (*Oryza sativa*)	Rice Genome Annotation Project v6.1
**Castor bean** (*Ricinus communis*)	TIGR v0.1
**Potato** (Solanum *tuberosum*)	SOL Genomics Network v4 and The International Potato Genome Sequencing Consortium (PGSC) v3.4
**Turnip** (*Brassica rapa*)	Phytozome v9.0
**Cassava** (*Manihot esculenta)*	Phytozome v7.0

Table S2. Comparison of pre-MeRecon (blue) and CassavaCyc (yellow) cassava carbon assimilation pathways

Sub-metabolisms	EC numbers	Reactions	Proteins
**Calvin cycle**	pre-MeRecon (21) CassavaCyc (20)<br>1 20	pre-MeRecon (23) CassavaCyc (20)<br>3 20	pre-MeRecon (158) CassavaCyc (146)<br>29 129 17
**Sucrose biosynthesis**	pre-MeRecon (15) CassavaCyc (9)<br>6 9	pre-MeRecon (16) CassavaCyc (8)<br>8 8	pre-MeRecon (79) CassavaCyc (60)<br>31 48 12
**Starch biosynthesis**	pre-MeRecon (13) CassavaCyc (10)<br>3 10	pre-MeRecon (14) CassavaCyc (8)<br>6 8	pre-MeRecon (106) CassavaCyc (89)<br>28 78 11
**Respiration**	pre-MeRecon (36) CassavaCyc (27)<br>9 27	pre-MeRecon (40) CassavaCyc (29)<br>11 29	pre-MeRecon (325) CassavaCyc (292)<br>101 224 68
**Amino acid biosynthesis**	pre-MeRecon (76) CassavaCyc (64)<br>14 62 2	pre-MeRecon (89) CassavaCyc (75)<br>16 73 2	pre-MeRecon (304) CassavaCyc (241)<br>110 194 47
**Cell wall biosynthesis**	pre-MeRecon (6) CassavaCyc (3)<br>3 3	pre-MeRecon (6) CassavaCyc (3)<br>3 3	pre-MeRecon (91) CassavaCyc (44)<br>68 23 21
**Fatty acid biosynthesis**	pre-MeRecon (12) CassavaCyc (6)<br>6 6	pre-MeRecon (35) CassavaCyc (19)<br>16 19	pre-MeRecon (65) CassavaCyc (34)<br>40 25 9
**Nucleotide biosynthesis**	pre-MeRecon (34) CassavaCyc (31)<br>4 30 1	pre-MeRecon (51) CassavaCyc (48)<br>4 47 1	pre-MeRecon (146) CassavaCyc (129)<br>43 103 26

Table S5. List of 35 enzymes (EC number) in MeRecon involved in multiple reactions. NUC - nucleotide biosynthesis, FAT - fatty acid biosynthesis, AMI - amino acid biosynthesis, CAL - Calvin cycle, RES - respiration, SUC - sucrose biosynthesis, and STA - starch biosynthesis

EC numbers	Sub-metabolisms	Related reactions (KEGG reaction IDs)	Number of the reactions
2.7.4.6	NUC	R00156, R00330, R00570, R01137, R01857, R02093, R02326, R02331	8
1.17.4.1	NUC	R02017, R02018, R02019, R02024	4
3.6.1.5	NUC	R00155, R00159, R00514, R00569	4
2.7.4.14	NUC	R00158, R00512	2
2.7.4.3	NUC	R00127, R01547	2
3.6.1.15	NUC	R00159, R00569	2
4.3.2.2	NUC	R01083, R04559	2
6.3.4.2	NUC	R00571, R00573	2
1.1.1.100	FAT	R04533, R04534, R04536, R04543, R04566, R04953, R04964	7
1.3.1.-	FAT	R04429, R04724, R04955, R04958, R04961, R04966, R04969	7
1.3.1.9	FAT	R04429, R04724, R04955, R04958, R04961, R04966, R04969	7
4.2.1.-	FAT	R04428, R04535, R04537, R04544, R04568, R04954, R04965	7
2.3.1.41	FAT	R04726, R04952, R04957, R04960, R04963, R04968	6
2.3.1.180	FAT	R01624, R04355	2
6.4.1.2	FAT	R00742, R04386	2
1.1.1.86	AMI	R04440, R05068, R05069, R05071	4
2.2.1.6	AMI	R00014, R04672, R04673	3
2.6.1.42	AMI	R01090, R01214, R02199	3
1.1.1.23	AMI	R01163, R03012	2
2.6.1.1	AMI	R00355, R00734	2
2.6.1.9	AMI	R00734, R03243	2
4.1.3.27	AMI	R00985, R00986	2
4.2.1.20	AMI	R00674, R02722	2
4.2.1.33	AMI	R03968, R04001	2
4.2.1.9	AMI	R04441, R05070	2
2.2.1.1	CAL	R01641, R01830	2
1.1.1.42	RES	R00268, R01899	2
1.2.4.1	RES	R00014, R03270	2
1.2.4.2	RES	R00621, R03316	2
4.1.1.1	RES	R00014, R00755	2
4.2.1.3	RES	R01325, R01900	2
3.2.1.1	SUC	Reaction ID 1*, Reaction ID 3*	2
4.1.2.13	CAL/RES	R01070, R01829	2
2.7.1.40	CAL/RES/NUC	R00200, R00430, R01138, R01858	4
5.3.1.9	STA/SUC	R02739, R02740, R03321	3

* KEGG reaction IDs were not dictated for these reactions.
